# Transforming Growth Factor Beta Promotes Inflammation and Tumorigenesis in Smad4‐Deficient Intestinal Epithelium in a YAP‐Dependent Manner

**DOI:** 10.1002/advs.202300708

**Published:** 2023-06-01

**Authors:** Liansheng Liu, Yalong Wang, Shicheng Yu, Huidong Liu, Yehua Li, Shan Hua, Ye‐Guang Chen

**Affiliations:** ^1^ Guangzhou Institutes of Biomedicine and Health University of Chinese Academy of Sciences Chinese Academy of Sciences Guangzhou 510530 China; ^2^ Guangzhou Laboratory Guangzhou 510700 China; ^3^ The State Key Laboratory of Membrane Biology Tsinghua‐Peking Center for Life Sciences School of Life Sciences Tsinghua University Beijing 100084 China; ^4^ Center for Life Sciences School of Life Sciences Yunnan University Kunming 650500 China; ^5^ Jiangxi Medical College Nanchang University Nanchang 330031 China

**Keywords:** colitis‐associated cancer, inflammatory bowel disease, Smad family member 4, transforming growth factor beta, YAP/TAZ

## Abstract

Transforming growth factor beta (TGF‐*β*), a multifunctional cytokine, plays critical roles in immune responses. However, the precise role of TGF‐*β* in colitis and colitis‐associated cancer remains poorly defined. Here, it is demonstrated that TGF‐*β* promotes the colonic inflammation and related tumorigenesis in the absence of Smad family member 4 (Smad4). *Smad4* loss in intestinal epithelium aggravates colitis and colitis‐associated neoplasia induced by dextran sulfate sodium (DSS) and azoxymethane/dextran sulfate sodium (AOM/DSS), leading to over‐activated immune responses and increased TGF‐*β*1 levels. In *Smad4*‐deficient organoids, TGF‐*β*1 stimulates spheroid formation and impairs intestinal stem cell proliferation and lineage specification. YAP, whose expression is directly upregulated by TGF‐*β*1 after *Smad4* deletion, mediates the effect of TGF‐*β*1 by interacting with Smad2/3. Attenuation of YAP/TAZ prevents TGF‐*β*1‐induced spheroid formation in *Smad4* ^*−* */* *–*^ organoids and alleviates colitis and colitis‐associated cancer in *Smad4*‐deficient mice. Collectively, these results highlight an integral role of the TGF‐*β*/Smad4 axis in restraining intestinal inflammation and tumorigenesis and suggest TGF‐*β* or YAP signaling as therapeutic targets for these gastrointestinal diseases intervention.

## Graphical abstract

*Smad4* loss in intestinal epithelium aggravates colitis and colitis‐associated neoplasia induced by dextran sulfate sodium (DSS) and azoxymethane/dextran sulfate sodium (AOM/DSS), leading to over‐activated immune responses and increased TGF‐*β*1 levels. This effect is achieved by the Smad2/3‐YAP complex‐induced expression of pro‐inflammatory genes. These results highlight an integral role of the TGF‐*β*/Smad4 axis in restraining intestinal inflammation and tumorigenesis.

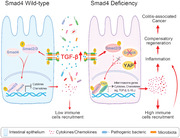

## 1 Introduction

Inflammatory bowel disease (IBD), including Crohn's disease (CD) and ulcerative colitis (UC), is a chronic and relapsing inflammatory disorder of the gastrointestinal tract characterized by continual pain, diarrhea, and bloody stools.^[^ ^1^, ^2^ ^]^ Despite its idiopathic nature, invasion and colonization of pro‐inflammatory bacteria and deficiencies in anti‐inflammatory bacteria are thought to correlate with and potentially contribute to IBD.^[^ ^3^, ^4^ ^]^ In addition, a compromised intestinal barrier may act as a predisposing factor that drives IBD progression, and facilitate the recruitment of immune cells in lamina propria to defense an excessive load of bacteria, resulting in hyper‐activated immune responses to invaded pathogens.^[^ ^5^, ^6^ ^]^ Genome‐wide association studies have identified a variety of predisposing genetic risk loci shared between UC and CD,^[^ ^7^, ^8^, ^9^ ^]^ implicating that a common circuit may underlie the pathogenesis of these diseases.

Increased IBD incidences pose significant health risks of developing colitis‐associated cancer (CAC), which approximately accounts for 10–15% of annual deaths in IBD patients.^[^ ^10^, ^11^, ^12^, ^13^ ^]^ The impaired intestinal barrier is susceptible to repetitive injuries, resulting in sustained inflammation, compensatory regeneration and finally hyperplasia.^[^ ^14^ ^]^ CAC can also arise from sequential mutation events, including those occurred in Wnt and transforming growth factor beta (TGF‐*β*) signaling components.^[^ ^15^, ^16^ ^]^ Importantly, *TP53* mutations are regarded as an early detrimental genetic event in CAC.^[^ ^17^ ^]^ Despite recent evidence suggesting that CAC is potentially initiated from an inflammatory environment and is strongly promoted by gut microbiota and impaired immune system,^[^ ^18^, ^19^, ^20^ ^]^ the molecular basis for the pathogenesis of CAC is still poorly understood.

TGF‐*β* is a key regulator that maintains tissue homeostasis in nearly all organs including the intestine. Emerging evidence has linked the pathogenesis of IBD with dysregulated TGF‐*β* signaling that affects mucosal immune reactions, immunomodulation of commensal bacterial strains and homeostasis of the epithelium.^[^ ^21^ ^]^ Mice with dominant‐negative *Tgfbr2* expressed in macrophages exhibit exacerbated colitis in response to DSS administration.^[^ ^22^ ^]^ Meanwhile, deficiencies in Smad family member 4 (*Smad4*) in T cells or the intestinal epithelium lead to spontaneous adenoma formation accompanied by elevated production of pro‐inflammatory cytokines and activated Wnt signaling.^[^ ^23^, ^24^ ^]^ High expression of *Smad7*, a negative regulator of TGF‐*β* signaling, in lamina propria mononuclear cells promotes inflammatory response in mice.^[^ ^25^ ^]^ Besides, mice bearing conditional loss of *Smad4* in epithelial cells in multiple endodermal organs display an upregulated expression of pro‐inflammatory genes, altered level of tight junction proteins and barrier functions, as well as promoted epithelial CCL20 signaling to CCR6^+^ immune cells, being predisposed to CAC.^[^ ^26^, ^27^, ^28^ ^]^ However, how the integrity of the TGF‐*β*/Smad4 axis in the intestinal epithelial compartment restrains colitis and CAC development remains obscure.

In this study, we show that *Smad4* deletion in mouse intestinal epithelium promotes intestinal inflammation and hyperplasia in chemically induced colitis and CAC. During this process, niche inflammatory signals are enhanced with increased TGF‐*β*1 levels. We find that TGF‐*β* signaling, in the absence of *Smad4*, remodels the cellular architecture of intestinal epithelium and provokes inflammatory responses by targeting *YAP*. Furthermore, genetic attenuation of YAP/TAZ activity alleviates dextran sulfate sodium (DSS)‐induced colitis and azoxymethane/dextran sulfate sodium (AOM/DSS)‐induced colorectal cancer in *Smad4*‐knockout mice. Collectively, our results uncover a novel mechanism in which *Smad4*‐independent TGF‐*β* signaling mediates colitis and CAC development via YAP and may provide therapeutic insights into the treatment of these gastrointestinal diseases.

## 2 Results

### 2.1 Inactivation of Smad4 in Intestinal Epithelial Cells Aggravates Dextran Sulfate Sodium‐Induced Colitis

To investigate the role of *Smad4* in intestinal inflammation, we first generated conditional, intestinal epithelium‐specific *Smad4* knockout mice (*Villin^CreER^*;*Smad4^fl/fl^*) (Figure S1a,b, Supporting Information). Surprisingly, we observed minimal effects on the small intestinal length and mouse body weight upon *Smad4* deletion (Figure S1c,d, Supporting Information). *Smad4^−/−^* mice displayed negligible morphological differences in intestinal barrier architecture compared with their wildtype (WT) littermates (Figure S1e, Supporting Information). However, *Smad4* deficiency led to a slightly expanded number of Olfm4^+^ stem cells (Figure S1f, Supporting Information), possibly due to impaired BMP signaling that would otherwise restrict the self‐renewal and proliferation of intestinal stem cells (ISCs).^[^ ^29^ ^]^ In contrast, the number of Muc2^+^ goblet cells as well as Paneth cells showed no significant changes (Figure S1f, Supporting Information). Nevertheless, *Smad4*‐deficient intestinal organoids exhibited normal growth (Figure S1g, Supporting Information). These results suggest that *Smad4* is largely dispensable for intestinal epithelium homeostasis in the early stages.

**Figure 1:**
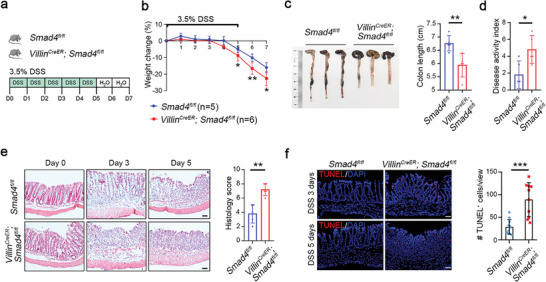
*Smad4*‐deficient mice are sensitive to DSS‐induced colitis. a) Scheme of the DSS‐induced colitis model in *Villin^CreER^*;*Smad4^fl/fl^* mice and their control littermates. b) The body weight change, gross morphological change, and c) colon length of *Smad4*‐deficient mice and the control littermates were assessed after feeding with 3.5% DSS in drinking water for 5 days, followed by a switch to normal water drinking for 2 days. Each genotype was represented by 5 or 6 biological replicates. d) The disease activity index of both control and *Smad4*‐deficient mice was monitored post DSS treatment, with a sample size of 3 mice for each genotype. e) The distal colon sections were subjected to H&E staining, and histology scores were obtained for both control and *Smad4*‐deficient mice after DSS treatment, with a sample size of more than 3 mice for each genotype at each time point. Scale bars: 50 µm. f) TUNEL staining was performed, and the TUNEL^+^ cells were quantified (right) in colonic sections obtained from both WT and *Smad4*‐deficient mice post DSS treatment. *n* = 3 per genotype. Scale bars: 50 µm. Data are pooled from three independent experiments in (b)–(f) and presented as means ± SD. Statistical significance was determined by unpaired, two‐tailed Student's *t*‐test. *p < 0.05, **p < 0.01, and ***p < 0.001.

We next challenged *Smad4* conditional knockout mice and WT littermates with 3.5% DSS, a chemical drug that can induces experimental colitis (**Figure** **1**a). Notably, severer colitis phenotypes, characterized by relatively more body weight loss, short colon, and higher clinical disease activity index (DAI) score, were observed in *Smad4*‐deficient mice (Figure 1b–d). In line with this, the pro‐inflammatory NF‐*κ*B and STAT3 signaling were enhanced while p38 signaling was slightly activated (Figure S2a,b, Supporting Information). Moreover, time‐course histological analysis revealed a disrupted structure and higher histological scores in colonic mucosal barrier in *Smad4^−/−^* mice after DSS administration (Figure 1e). Since cell death is thought to be a major contribution to the collapse of intestinal epithelial integrity,^[^ ^30^ ^]^ we then assessed cell survival in the DSS‐treated mice. Compared with WT littermates, the number of TUNEL‐positive apoptotic cells in *Smad4*‐deficient mice was dramatically increased (Figure 1f). Correspondingly, PUMA*α*/*β*, a key mediator of epithelial cell death in colitis,^[^ ^31^ ^]^ and other apoptosis‐associated proteins, including Bax and cleaved caspase3, showed significantly upregulated expression in *Smad4^−/−^* colonic epithelial cells (Figure S2c, Supporting Information). Taken together, these results indicate that *Smad4* plays a critical role in attenuating colitis development in an epithelial cell‐autonomous manner.

### 2.2 Epithelial Smad4 Deficiency Promotes Azoxymethane/Dextran Sulfate Sodium‐Induced Colitis‐Associated Cancer

**Figure 2:**
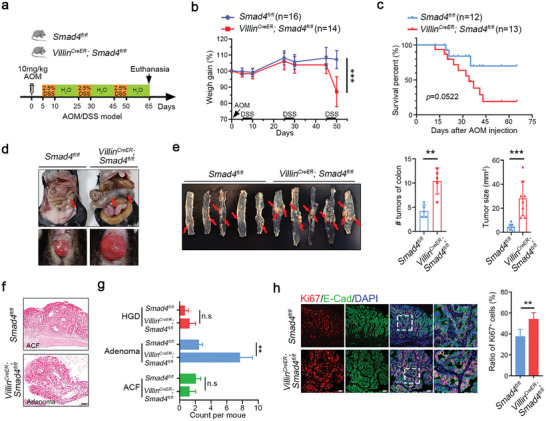
*Smad4* deficiency accelerates AOM/DSS‐induced colitis‐associated tumorigenesis. a) Scheme of the AOM/DSS model for colitis‐associated cancer. b) The body weight changes and c) survival rate of the mice after AOM/DSS treatment are presented. Indicated biological replicates are indicated. d,e) Following AOM/DSS treatment, gross morphological images of the colons and colonic tumors were obtained, and the tumor number and size were measured. A sample size of *n* = 5 per genotype. The pathological area and manifestation of tumors in the colon are indicated by arrows. f) The colonic tumors after AOM/DSS treatment were subjected to H&E staining for histological examination and analysis. ACF: Aberrant crypt foci; *n* = 5 per genotype. Scale bar: 100 µm. g) Quantification of the number of tumor subtypes. ACF: Aberrant crypt foci; HGD: High‐grade dysplasia. *n* = 5 per genotype. h) Immunofluorescence of Ki67 and E‐cadherin in colonic tumor sections from the indicated mice. The quantitative results are shown on the right. Scale bar: 50 µm. Data are presented as means ± SD with statistical significance determined by a two‐tailed Student's *t*‐test unless otherwise indicated. *p < 0.05, **p < 0.01, and ***p < 0.01, n.s, no significance.

Given the strong inflammatory phenotypes observed in epithelial *Smad4*‐deficient mice treated with DSS and the predisposing role of inflammation in cancer development,^[^ ^32^ ^]^ we extended our investigation to determine whether *Smad4* plays a role in inflammation‐associated intestinal tumorigenesis. We intraperitoneally injected mice with azoxymethane (AOM) followed by three cycles of 2.5% DSS treatment to induce CAC (**Figure** **2**a). *Smad4^−/−^* mice displayed drastic body weight loss in the late time (Figure 2b), and showed a markedly reduced lifespan compared to their control counterparts (Figure 2c). Increased tumor number and mass were observed in *Smad4*‐deficient mice (Figure 2d,e). Besides, histopathological analysis of *Smad4*‐deficient colon sections revealed severer epithelial barrier disruption, an increased number of adenomas, and the presence of cribriform patterns associated with high‐grade dysplasia (Figure 2f,g). Importantly, the *Smad4^−/−^* tumors showed a malignant proliferative capacity (Figure 2h). These results together corroborate the notion that *Smad4* deficiency predisposes mice to CAC.

### 2.3 Loss of Smad4 Provokes Hyper‐Reactive Immune Responses in the Intestinal Mucosa after DSS Treatment

**Figure 3:**
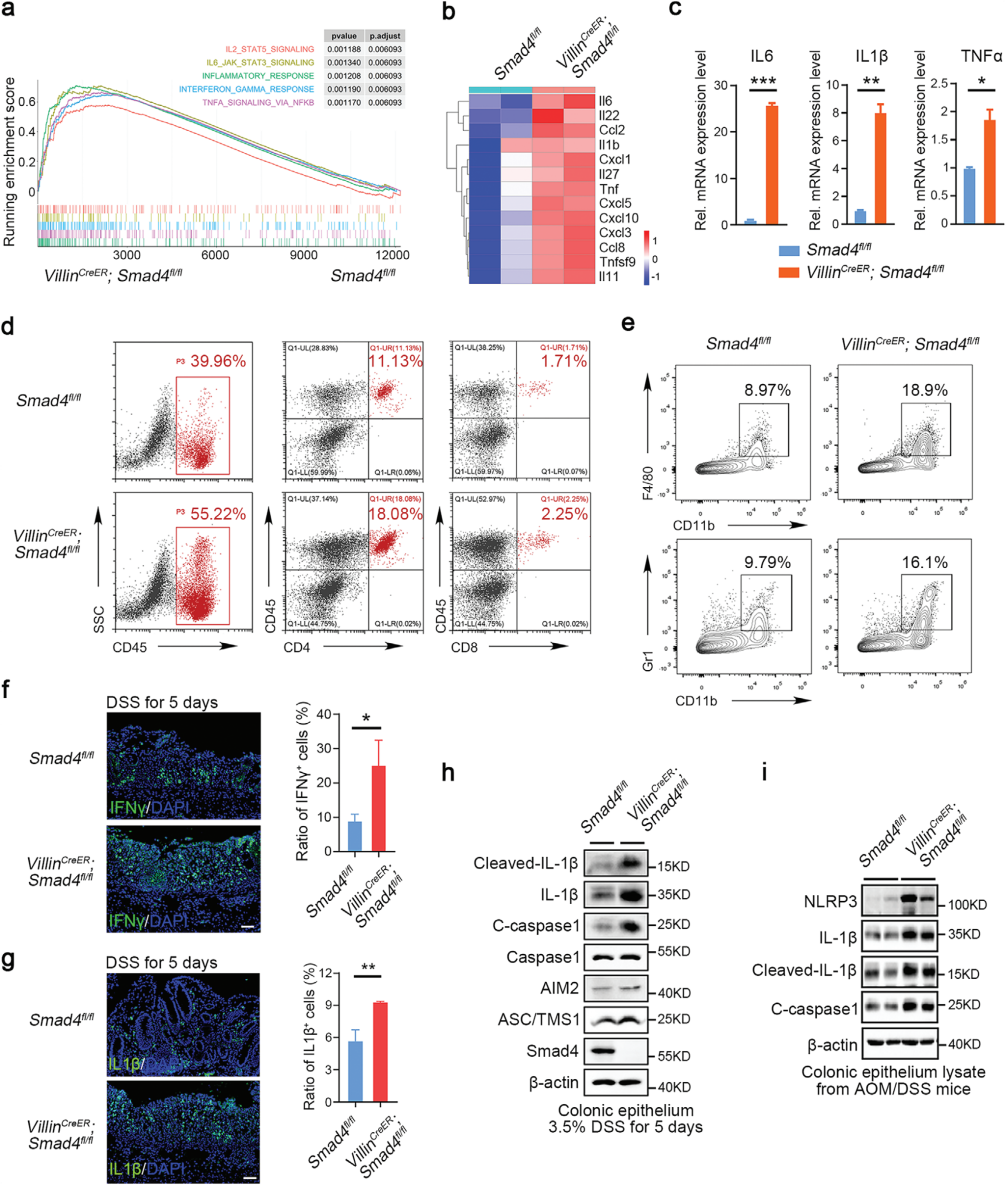
*Smad4* deficiency leads to hyper‐reactive mucosal immunity. a) The genes associated with inflammatory response and other immune‐regulating pathways were analyzed using GSEA in DSS‐treated *Smad4^fl/fl^* and *Villin^CreER^*;*Smad4^fl/fl^* mice. b) Heatmap shows the expression of the indicated inflammatory cytokines in the colonic epithelium from DSS‐treated *Smad4^fl/fl^* and *Villin^CreER^*;*Smad4^fl/fl^* mice. *n* = 2 mice for each genotype. c) The mRNA expression of pro‐inflammatory cytokines (IL‐6, IL‐1*β*, and TNF‐*α*) in the colonic epithelium of control and *Smad4*‐deficient mice at day 7 following DSS treatment. d,e) The colon‐infiltrated immune cells of DSS‐treated *Smad4^fl/fl^* and *Villin^CreER^*;*Smad4^fl/fl^* mice (*n* = 3 mice per group) at day 5 following DSS treatment were analyzed using flow cytometry. SSC, side scatter. f,g) Immunofluorescence staining of IFN*γ* and IL‐1*β* in the colon of *Smad4^fl/f^* and *Villin^CreER^*;*Smad4^fl/fl^* mice following DSS treatment for 5 days. Quantitative analysis was shown on the right. The sample size for each group was *n* = 3 mice. Scale bar: 50 µm. h) Immunoblotting analysis of inflammasome proteins in the colonic epithelium of the indicated mice after DSS treatment for 5 days. i) Immunoblotting analysis of inflammasome proteins in the colonic epithelium of mice treated with AOM/DSS. The sample size for each group was *n* = 2 mice. Statistical analysis is performed using independent *t*‐test for (c), (f), and (g). *p < 0.05, **p < 0.01, ***p < 0.001.

*Smad4* has long been documented as a tumor suppressor in the development of colorectal cancer, owing to its key role in mediating TGF‐*β* signaling to block proliferation or induce apoptosis.^[^ ^23^, ^33^, ^34^ ^]^ Our foregoing results suggest that, in addition to its cytostatic effect, *Smad4* may retard inflammation‐induced intestinal tumorigenesis by restricting the inflammatory processes. We therefore examined whether immune responses were perturbed in *Smad4*‐deficeint mice subject to DSS‐induced colitis. The gene set enrichment analysis (GSEA) revealed that many genes associated with multiple pro‐inflammatory signaling, immune cell proliferation and epithelial cell apoptosis were upregulated upon *Smad4* deletion (**Figure** **3**a and Figure S3a, Supporting Information). In particular, there was a remarkably increased expression of genes encoding pro‐inflammatory cytokines or chemokines in *Smad4*‐deficient mice treated with DSS, such as *Il* *‐* *6*, *Il* *‐* *1*β, and *Tnf*α (Figure 3b,c). Notably, there was an increase in the proportion of infiltrated immune cells (CD45^+^), CD4^+^ and CD8^+^ T cells, macrophages (CD11b^+^F4/80^+^), and neutrophils (CD11b^+^Gr1^+^) in *Smad4^−/−^* mice during colitis compared with those in WT mice (Figure 3d,e), which was accompanied by enhanced IFN*γ* levels (Figure 3f). Besides, the expression of IL7R and IL17R in the inflamed area was also upregulated (Figure S3b, Supporting Information). Macrophages are highly plastic and heterogeneous and can be polarized into the pro‐inflammatory M1 type or the anti‐inflammatory M2 type depending on the contextual stimuli.^[^ ^35^ ^]^ In *Smad4^−/−^* mice, the F4/80^+^ macrophages showed an increased number with elevated expression of M1 markers (CD80 and CD86) but declined expression of M2 markers (CD204 and CD206) (Figure S3c,d, Supporting Information).

Il‐1*β*, which is an important component of the NLRP3 inflammasome, a cytosolic protein complex of the innate immune system that regulates inflammation, pyroptosis, and gut homeostasis,^[^ ^36^ ^]^ displayed a pronounced elevation in *Smad4*‐deleted mice following DSS treatment (Figure 3g). To further verify that *Smad4* deficiency plays an epithelial‐intrinsic role in activating the inflammasome pathway, we isolated the colonic epithelium from DSS‐challenged *Smad4*‐deficient mice and performed quantitative PCR (qPCR) analysis. A collection of typical inflammasome genes, such as *Nlrp3*, *Il* *‐* *1*β, and *Il‐18*, showed an increased expression (Figure S3e, Supporting Information). Upregulated expression of these inflammasome components, except for the scaffold protein ASC and the receptor AIM2, was also confirmed at the protein level in both colonic epithelium and tumors from DSS‐ and AOM/DSS‐treated *Smad4*‐deficient mice, including Nlrp3, Il‐1*β*, and cleaved caspase‐1 (Figure 3h,i and Figure S3f,g, Supporting Information). These data indicate that epithelial depletion of *Smad4* intensifies immune responses during colitis, leading to deleterious inflammation.

### 2.4 Altered Transforming Growth Factor Beta Responses in Smad4^−/−^ Intestinal Epithelium

**Figure 4:**
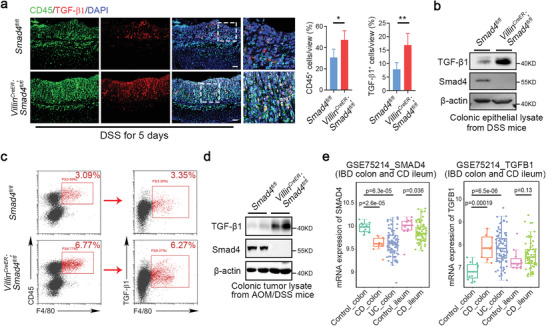
Upregulation of TGF‐*β*1 in Smad4‐deficient intestinal epithelium. a) Immunofluorescence staining of colon sections using anti‐CD45 and anti‐TGF‐*β*1 antibodies. The quantification of TGF‐*β*1^+^ and CD45^+^ cells was presented on the right. Three mice were used for each group. Scale bar: 50 µm. b) Immunoblotting analysis of TGF‐*β*1 protein in the colonic epithelium of DSS‐treated mice. c) Flow cytometric analysis of colon‐infiltrated TGF‐*β*1^+^F4/80^+^ macrophages in DSS‐treated *Villin^CreER^*; *Smad4^fl/fl^* and littermate control *Smad4^fl/fl^* mice. d) Immunoblotting analysis of TGF‐*β*1 protein in the colonic tumor from mice treated with AOM/DSS. 2 mice per group. e) Box plots were generated to show the mRNA expression of *S* *MAD4* and *TGFB* *1* in healthy and inflammatory bowel disease (IBD) specimens using dataset GSE75214. The middle line in the box plots represents the median, while the whiskers indicate the minimum‐to‐maximum range of the data distribution. Error bars show means ± SD. Statistical significance is determined by a two‐tailed Student's *t*‐test. *p < 0.05, **p < 0.01.

Considering that TGF‐*β* signaling negatively regulates immune activities and is critical to immune suppression,^[^ ^37^ ^]^ we examined TGF‐*β*1 expression in the DSS‐treated *Smad4*‐deficient intestine. We observed a pronounced increase in the proportion and number of infiltrated CD45^+^ leukocytes and TGF‐*β*1^+^ cells across the colonic epithelium in *Smad4^−/−^* mice (Figure S4a,b, Supporting Information), which appeared more striking when the mice were challenged with DSS for 5 days (**Figure** **4**a). We also observed an increased TGF‐*β*1^+^ZO‐1^+^ cells in *Smad4^−/−^* mice after DSS treatment, indicating a specific role of *Smad4* in regulating TGF‐*β*1‐dependent inflammation in the intestine epithelial cells (Figure S4c, Supporting Information). Consistent with these results, TGF‐*β*1 expression was significantly upregulated at both the protein and mRNA levels (Figure 4b and Figure S4d,e, Supporting Information). TGF‐*β* has been documented in regulating immune responses.^[^ ^35^ ^]^ By evaluating the proportion of TGF‐*β*1^+^ macrophages in colon lamina propria, we found an elevated TGF‐*β*1^+^F4/80^+^ macrophages in *Smad4^−/−^* mice compared with control littermates after DSS challenge (Figure 4c), suggesting that TGF‐*β*1 derived from macrophages might also play a role in intestinal pathogenesis after *Smad4* loss. Of note, the abundance of TGF‐*β*1 was also increased in intestinal tumors from *Smad4^−/−^* mice treated with AOM/DSS (Figure 4d). These results prompted us to ask whether there is a correlation between enhanced TGF‐*β*1 expression and reduced Smad4 levels in IBD patients. Through analyzing the gene expression omnibus (GEO) Datasets, we confirmed that increased *TGFB* *1* levels were coincided with decreased *SMAD4* in gut biopsies from IBD patients (Figure 4e and Figure S4f, Supporting Information).

**Figure 5:**
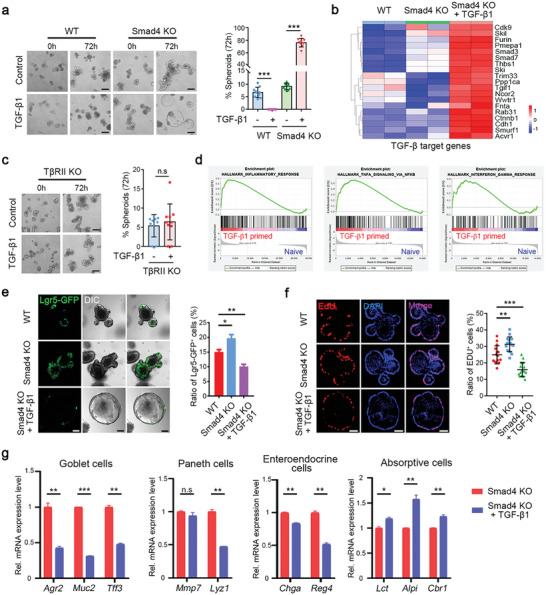
TGF‐*β* impairs ISC proliferation and differentiation. a) The organoids originated from the small intestine of both WT and *Smad4* KO mice were treated with or without 0.5 nM TGF‐*β*1 for 72 h. The spheroids ratio was quantified on the right. Scale bar: 100 µm. b) Heatmap illustrates the expression of TGF‐*β* target genes in organoids derived from the small intestine of WT, *Smad4* KO specimens, and TGF‐*β*1‐treated *Smad4* KO specimens. Each genotype was represented by two biological replicates. c) Bright field images of WT or T*β*RII KO organoids with or without 0.5 nM TGF‐*β*1 stimulation for 72 h. The quantitation is shown on the right. Scale bar: 100 µm. d) GSEA analysis of the enriched signaling pathways in *Smad4*‐deficient organoids after TGF‐*β*1 treatment. e) Representative images and the quantification of Lgr5‐GFP^+^ cells in the indicated organoids. Scale bar: 100 µm. f) EdU staining to evaluate the proliferation rate of the specified organoids, followed by statistical analysis of the percentage of EdU^+^ cells. Scale bar: 100 µm. g) RT‐qPCR analysis of the marker genes expression of the differentiated cells in the TGF‐*β*1‐treated *Smad4^−/−^* organoids. Data are presented as means ± SD. Statistical significance determined by a two‐tailed Student's *t*‐test. *p < 0.05, **p < 0.01, ***p < 0.001, n.s, no significance.

Given an upregulated intestinal TGF‐*β*1 expression in *Smad4*‐deficent mice treated with DSS, we then attempted to decipher the role of TGF‐*β*1 in the intestinal epithelium using intestinal organoids. TGF‐*β*1 treatment led to death of WT organoids likely due to the cytostatic effect of TGF‐*β*/Smad4 signaling. However, TGF‐*β*1 did not induce cell death in *Smad4^−/−^* intestinal organoids. Instead, it triggered a rapid morphology change from budding to spheroid structures (**Figure** **5**a and Figure S5a, Supporting Information). No apparent spheroid formation was observed in WT and *Smad4^−/−^* organoids treated with BMP4, suggesting this effect was TGF‐*β*1‐specific (Figure S5b, Supporting Information). Interestingly, TGF‐*β*1 could still activate the expression of many target genes of canonical TGF‐*β* signaling in *Smad4^−/−^* organoids (Figure 5b). The TGF‐*β*1‐induced morphological change in *Smad4^−/−^* organoids depended on TGF‐*β* receptors as genetic disruption of TGF*β*RII completely abolished spheroid formation (Figure 5c). Importantly, the TGF‐*β*1‐upregulated genes were also involved in inflammatory responses characterized by the activation of TNF*α* and IFN*γ* signaling, and short‐time treatment of TGF‐*β*1 promoted the expression of pro‐inflammatory cytokine and chemokine genes in *Smad4^−/−^* organoids (Figure 5d and Figure S5c, Supporting Information), which recapitulated the in vivo immune response.

To further investigate whether TGF‐*β*1 regulates cell fate determination in the absence of *Smad4*, we first assessed ISCs in *Smad4*‐deficient organoids. TGF‐*β*1 reduced Lgr5‐GFP^+^ stem cells (Figure 5e and Figure S6a, Supporting Information) and attenuated the expression of stem cell markers, which was reversed by the TGF*β*RI inhibitor SB431542 (Figure S6b, Supporting Information). The cell division in *Smad4*‐deficient organoids was impeded after treated with TGF‐*β*1 (Figure S6c, Supporting Information). In addition, we also observed decreased cell proliferation as labeled by EdU (5‐ethynyl 2’‐deoxyuridine) or Ki67 in *Smad4^−/−^* organoids (Figure 5f and Figure S6d, Supporting Information). Moreover, TGF‐*β*1 impaired the expression of Mucin 2 (goblet cell marker), lysozyme 1 (Paneth cell marker), and chromogranin‐A (enteroendocrine cell marker) in *Smad4*‐knockout organoids (Figure 5g and Figure S6e–g, Supporting Information), indicating an impaired differentiation of these cell types. However, the Alpi^+^ absorptive cell differentiation was enhanced (Figure 5g and Figure S6h, Supporting Information). Altogether, these results suggest that TGF‐*β*1 impairs stemness, cell proliferation, and secretory cell differentiation in *Smad4*‐deficient intestinal organoids.

### 2.5 YAP/TAZ Mediates the Effect of TGF‐*β*1 in Smad4‐Deficient Organoids

**Figure 6:**
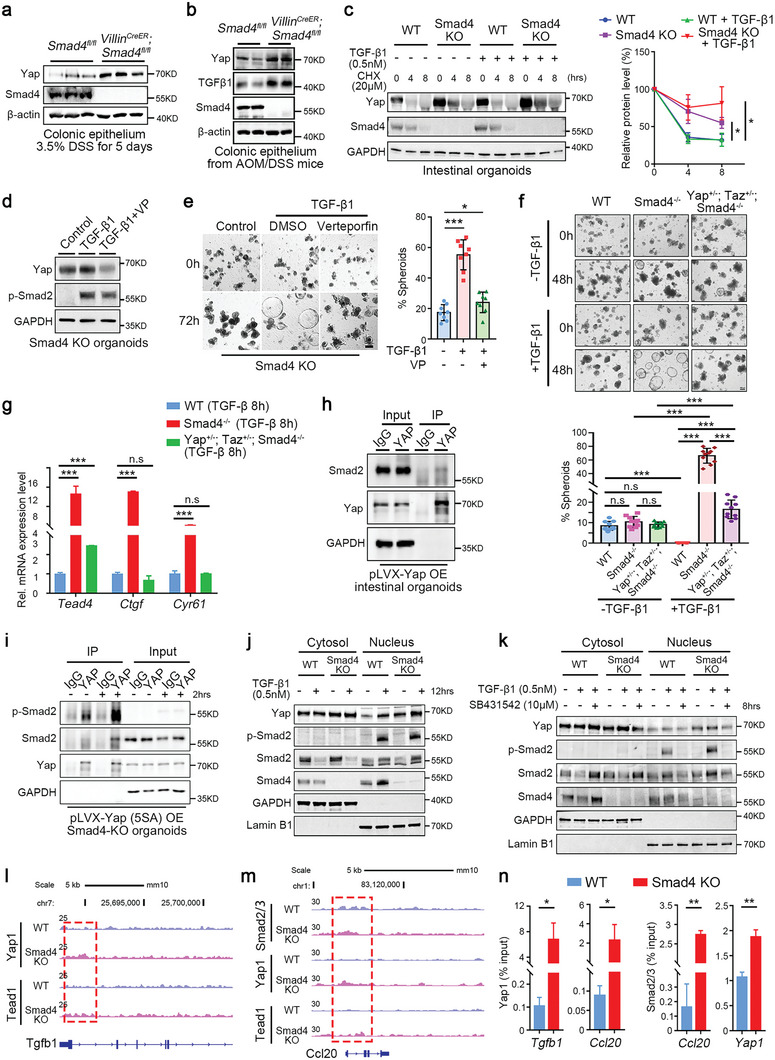
YAP mediates the TGF‐*β*1‐induced spheroid formation. a,b) Immunoblotting of the specified proteins in the colonic epithelium of mice challenged with a) 3.5% DSS for 5 days (*n* = 3 mice for each group) or b) with AOM/DSS (*n* = 2 mice for each group). c) The half‐life of YAP protein was assessed in WT and *Smad4* KO organoids after the indicated treatments. Quantitation is shown on the right. d) Immunoblotting of YAP and phosphorylated Smad2 in *Smad4*‐KO organoids following TGF‐*β*1 treatment or in combination with Veterporfin (VP). e) Bright field images of *Smad4*‐KO organoids after TGF‐*β*1 and VP treatment. The ratio of spheroids in the total organoids were quantitated on the right. *n* = 3 biological replicates for each timepoint. Scale bar: 50 µm. f) Bright field images of WT, *Smad4^−/−^*, and *Smad4^−/−^/Yap^+/−^/Taz^+/−^* organoids with or without TGF‐*β*1 treatment. The ratio of spheroids in the total organoids were quantitated in the right. Scale bar: 50 µm. g) RT‐qPCR analysis of YAP target genes in intestinal organoids obtained from the indicated mice following TGF‐*β*1 treatment for 8 h. h) Interaction of ectopically expressed YAP with endogenous Smad2 as shown by co‐immunoprecipitation. FLAG‐tagged YAP was overexpressed in WT intestinal organoids. i) Co‐immunoprecipitation revealed the interaction between endogenous Smad2 and ectopically expressed YAP(5SA) in intestinal organoids following treatment with or without 0.5 nM TGF‐*β*1 for 2 h. j) Immunoblotting analysis of the nuclear distribution of the indicated proteins in response to TGF‐*β*1 stimulation in both WT and *Smad4* KO organoids. k) Immunoblotting analysis of the nucleocytoplasmic shuttling of the indicated proteins in response to TGF‐*β*1 and SB431542 in both WT and *Smad4* KO organoids. l,m) Genomic views of Yap1, Smad2/3, and Tead1 ChIP enrichment at the promoters of the indicated genes in WT and *Smad4* KO organoids after TGF‐*β*1 stimulation for 12 h. n) ChIP‐qPCR was conducted on colonic epithelium from DSS‐treated *Smad4* KO and control littermates to validate Yap1 and Smad2/3 binding to the indicated sites. Data are presented as means ± SD with statistical significance calculated by a two‐ tailed Student's *t*‐test. *p < 0.05, **p < 0.01, ***p < 0.001, n.s, no significance.

To uncover the molecular mechanism underlying the observed effects of TGF‐*β*/Smad4 on colitis and CAC, we interrogated the available gene expression of the colonic tissues from CD and UC patients. Intriguingly, many YAP target genes were upregulated in both UC and CD patients (Figure S7a, Supporting Information). Analysis of the IBD patient database (GSE38713,^[^ ^38^ ^]^ GSE75214^[^ ^39^ ^]^) revealed a positive correlation between *YAP1* and *TGFB1* expression in these biopsies (Figure S7b, Supporting Information). GSEA analysis also revealed an upregulated expression of YAP signature genes in the DSS‐treated *Smad4^−/−^* mouse colon (Figure S7c, Supporting Information). Indeed, YAP was significantly increased in the DSS‐treated *Smad4^−/−^* colonic epithelium (**Figure** **6**a). Similarly, YAP and TGF‐*β*1 were concurrently elevated in the AOM/DSS‐induced *Smad4^−/−^* intestinal tumors (Figure 6b). Importantly, TGF‐*β*1 enhanced YAP expression and upregulated many YAP target genes in *Smad4^−/−^* organoids (Figure S7d, Supporting Information). The half‐life of YAP protein was prolonged in *Smad4^−/−^* organoids (Figure 6c).

To explore whether YAP mediates the TGF‐*β*1 effect on *Smad4^−/−^* organoids, we treated TGF‐*β*1‐stimulated organoids with Verteporfin (VP), a small molecule that disrupts the YAP‐TEAD interaction and therefore inhibits Hippo/YAP signaling‐regulated transcription.^[^ ^40^ ^]^ Of interest, the increased YAP level in *Smad4*‐decificient intestinal organoids upon TGF‐*β*1 treatment was reduced after simultaneous addition of VP (Figure 6d). As expected, the nuclear accumulation of YAP was reduced to a level comparable to that in the *Smad4*‐knockout control in TGF‐*β*‐induced spheroids following VP treatment (Figure S7e, Supporting Information). Furthermore, VP partially blocked the TGF‐*β*1‐induced spheroid formation and reversed the TGF‐*β*1‐enhanced expression of YAP target genes in *Smad4^−/−^* organoids, such as *Tead4*, *Ctgf*, and *Cyr61* (Figure 6e and Figure S7f, Supporting Information). Consistently, we also observed reduced spheroid formation and decreased expression of YAP target genes after TGF‐*β*1 exposure in *ex vivo* intestinal organoids derived from *Smad4^−/−^/Yap^+/−^/Taz^+/−^* mice (Figure 6f,g). In addition, ectopic expression of the human transcriptionally inactive mutant YAP(S94A) also inhibited the TGF‐*β*1‐induced spheroid formation (Figure S7g, Supporting Information). These data suggest that YAP acts downstream of TGF‐*β*1 in *Smad4*‐deficient intestinal organoids.

To further verify that YAP mediates TGF‐*β* signaling, we examined whether YAP physically interact with Smad proteins in intestinal epithelial cells. Co‐immunoprecipitation revealed a strong interaction between YAP and Smad2/3 but not Smad4, which could be potentiated by TGF‐*β*1 (Figure 6h and Figure S8a–c, Supporting Information). Furthermore, Smad2 interacted with ectopically expressed YAP(5SA) or YAP(S127A), two active mutant forms of YAP, and the interaction was enhanced by TGF‐*β*1 (Figure 6i and Figure S8d,e, Supporting Information). However, there was no direct association between Smad2/3 and YAP(S94A), a mutant defective in TEAD‐binding,^[^ ^41^ ^]^ implying that YAP‐TEAD interaction plays a role in the YAP‐Smad2/3 interaction (Figure S8f,g, Supporting Information). Moreover, Smad2/3 also interacted more efficiently with TAZ, a homolog of YAP, after TGF‐*β*1 treatment (Figure S8h,i, Supporting Information). Then, we determined the phosphorylation of Smad2/3 in mediating YAP subcellular localization in view of the enhanced nuclear shuttling of YAP upon TGF‐*β* exposure in *Smad4*‐deficient organoids (Figure 6j). A reduced YAP level was observed in the nucleus after pretreatment of the T*β*RI inhibitor SB431542 (Figure 6k), indicating that the TGF‐*β* receptor‐triggered phosphorylation of Smad2/3 plays a role in YAP activation. Together, these data suggest that YAP/TAZ interacts with Smad2/3 in response to TGF‐*β*1 and works in concert to confer their nuclear distribution and transcriptional potency.

As *Smad4* deficiency enhances inflammatory response, we then determined whether Smad2/3‐YAP interaction contributes to the expression of the inflammatory genes by performing chromatin immunoprecipitation (IP) analysis in colonic epithelium from DSS‐treated mice and TGF‐*β*1‐stimulated intestinal organoids. The activation of YAP signaling was validated in *Smad4*‐deficient intestinal epithelium as both of YAP1 and TEAD1, a co‐factor of YAP, were found in the YAP target genes such as *Ctgf* (Figure S8j, Supporting Information). Moreover, we observed significantly higher occupancy of YAP1 and TEAD1 in *Smad4*‐deficient epithelium than in the control in the *Tgfb1* gene (Figure 6l), consistent with the elevated expression of TGF‐*β*1 in *Smad4*‐deficient epithelium. In addition, enhanced binding of YAP1 and TEAD1 was found in key inflammasome genes such as *Nlrp6* and *Il18* upon *Smad4* knockout (Figure S8k,l, Supporting Information). Furthermore, binding of YAP1, TEAD1 and Smad2/3 to the promoter region of *Ccl20* and *Cxcl1* was elevated in *Smad4*‐deficient epithelium compared to WT controls (Figure 6m and Figure S8m, Supporting Information), supporting the notion that *Smad4* loss significantly promotes the transcription of key inflammatory genes. ChIP‐qPCR validation for selected loci confirmed higher YAP1, TEAD1, and Smad2/3 occupancy in *Smad4* KO mice (Figure 6n and Figure S8n, Supporting Information). Furthermore, higher Smad2/3 occupancy was found at the *Yap1* gene promoter in *Smad4* KO intestinal epithelium (Figure 6n). Collectively, these findings demonstrate the promoting role of Smad2/3/YAP signaling in inflammation upon *Smad4* loss.

### 2.6 Heterozygous Knockout of YAP/TAZ Suppresses Intestinal Inflammation and Tumorigenesis in Smad4‐Deficient Mice

**Figure 7:**
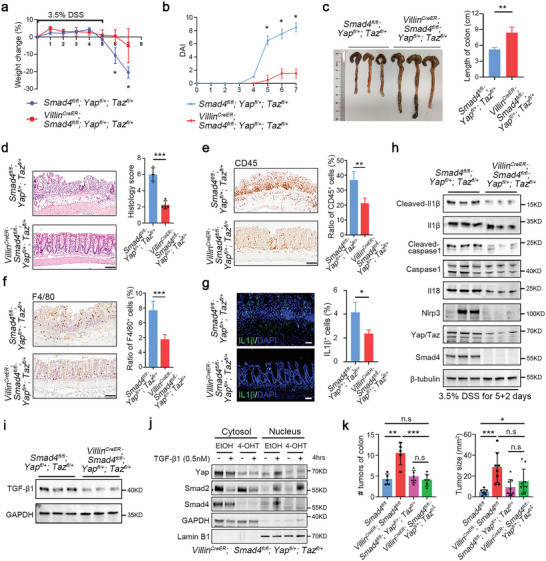
Reducing YAP/TAZ expression alleviates the progression of colitis and tumorigenesis in *Smad4*‐deficient mice. a) Body weight and b) disease activity index were monitored daily in littermate control and *Smad4^−/−^/Yap^+/−^/Taz^+/−^* mice following DSS treatment for 5 days. *n* = 5 mice per group. c) Representative gross images of colons from *Smad4^−/−^/Yap^+/−^/Taz^+/−^* and control littermates following DSS treatment for 5 days. Quantification of colon length is shown on the right. *n* = 3 mice per group. d) Histological images of colonic tissues from *Smad4^−/−^/Yap^+/−^/Taz^+/−^* and control littermates following DSS treatment, along with quantification of histological scores. The sample size for this analysis was *n* = 3 mice per group. Scale bar: 100 µm. Immunohistochemical analysis of e) CD45 and f) F4/80 in colon sections from *Smad4^−/−^/Yap^+/−^/Taz^+/−^* and control littermates following DSS treatment for 5 days. The quantitation is shown on the right. *n* = 3 mice per group. Scale bar: 100 µm. g) Immunostaining of IL‐1*β* in colon sections derived from *Smad4^−/−^/Yap^+/−^/Taz^+/−^* and control littermates following DSS treatment for 5 days. Quantification of IL‐1*β* ^+^ cells is presented on the right. *n* = 3 mice per group. Scale bar: 50 µm. h) Immunoblotting analysis of inflammasome proteins in the colonic epithelium of mice after DSS treatment for 5 days. *n* = 3 mice for each group. i) Immunoblotting of TGF‐*β*1 protein in the colonic epithelium of mice after DSS treatment for 5 days. The sample size for this analysis was *n* = 3 mice for each group. j) Immunoblotting analysis of the cytoplasmic/nuclear distribution of the indicated proteins in intestinal organoids derived from *Villin^CreER^;Smad4^fl/fl^;Yap^fl/+^;Taz^fl/+^* mice with or without 4‐hydroxytamoxifen treatment. k) Tumor number and size were quantified in the indicated mice treated with AOM/DSS to induce CAC (*n* = 5–6 mice per group). Data are presented as means ± SD with statistical analyses determined by two‐tailed Student's *t*‐test. *p < 0.05, **p < 0.01, ***p < 0.001, n.s, no significance.

Both YAP and TAZ mediate Hippo signaling and have functional redundancy in mammals.^[^ ^42^ ^]^ To examine the role of YAP in DSS‐induced colitis of *Smad4^−/−^* mice, we generated *Villin^CreER^*;*Smad4^fl/fl^*;*Yap^fl/+^*;*Taz^fl/+^* mice (*Smad4^−/−^/Yap^+/−^/Taz^+/−^*). Of note, heterozygous deletion of *Yap* and *Taz* had little effect as their intestine architecture and histological structures looked similar to WT mice (data not shown). *Smad4^−/−^/Yap^+/−^/Taz^+/−^* mice showed less body weight loss, lower clinical DAI and longer colon length (**Figure** **7**a–c). Consistently, 2‐month‐old *Smad4^−/−^/Yap^+/−^/Taz^+/−^* mice displayed reduced diarrhea and less rectal bleeding compared to their control littermates in the DSS‐induced colitis mice (data not shown). Histopathological examination verified that *Smad4^−/−^/Yap^+/−^/Taz^+/−^* mice had relatively intact tissue architecture with lower clinical histological scores in the DSS‐treated colon (Figure 7d). Besides, the colon exhibited less disrupted patterning of the tight junction protein ZO‐1 (Figure S9a, Supporting Information), and goblet cells were recovered (Figure S9b, Supporting Information), indicating that reduced expression of YAP/TAZ compensates the deteriorating effect of *Smad4* deficiency in colitis.

We then proceeded to examine whether heterozygous loss of YAP/TAZ can suppress intestinal inflammation in *Smad4*‐deficient mice. The proportion and number of infiltrated leukocytes, including monocytes/macrophages and T cells were decreased in the colon in DSS‐treated *Smad4^−/−^/Yap^+/−^/Taz^+/−^* mice compared to control littermates (Figure 7e,f and Figure S9c, Supporting Information). Consistently, IL1*β* ^+^ and IFN*γ* ^+^ cells as well as IL7R^+^ and IL17R^+^ cells all showed a significant reduction in the DSS‐treated *Smad4^−/−^/Yap^+/−^/Taz^+/−^* mice (Figure 7g and Figure S9d–f, Supporting Information). Heterozygous loss of YAP/TAZ also decreased the expression of TGF‐*β*1 and inflammasome‐associated proteins, such as cleaved Il‐1*β*, cleaved caspase‐1, Il18, and Nlrp3 (Figure 7h,i). In addition, ameliorated cell death was observed in the DSS‐treated *Smad4^−/−^/Yap^+/−^/Taz^+/−^* intestine (Figure S9g, Supporting Information). Notably, intestinal organoids derived from *Villin^CreER^*;*Smad4^fl/fl^*;*Yap^fl/+^*;*Taz^fl/+^* mice following 4‐hydroxytamoxifen addition also showed reduced nuclear distribution of YAP (Figure 7j). Furthermore, a reduced expression of some pro‐inflammatory cytokine and chemokine genes was found in *Smad4^−/−^/Yap^+/−^/Taz^+/−^* organoids comparing with those from *Smad4^−/−^* organoids (Figure S9h, Supporting Information). Furthermore, less tumors were found in *Smad4^−/−^/Yap^+/−^/Taz^+/−^* mice compared to *Smad4^−/−^* mice in the AOM/DSS model (Figure 7k). These results demonstrate that YAP/TAZ are a key mediator in colitis and tumorigenesis in *Smad4*‐deficient mice, and attenuation of YAP/TAZ activity can effectively prevent the pathological outcomes.

## 3 Discussion

**Figure 8:**
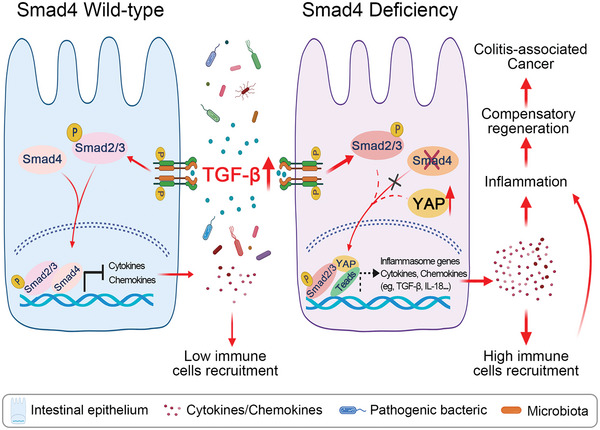
Schematic overview of the epithelium‐intrinsic TGF‐*β*/*Smad4* signaling in colitis formation and colitis‐associated tumorigenesis. In wild‐type intestinal epithelial cells, TGF‐*β* induces phosphorylation of Smad2/3, and the complex formation with *Smad4*. This Smad complex is then accumulated in the nucleus where it suppresses the production of inflammatory cytokines and chemokines. In *Smad4*‐deficient intestinal epithelial cells, the elevated TGF‐*β* and YAP expression potentiate the binding of phosphorylated Smad2/3 to YAP, and the resulting complex activates the expression of inflammasome genes, leading to immune cell recruitment and inflammation and thus accelerating the development of colitis and colitis‐associated cancer.

Intestinal inflammation is usually ascribed to hereditary susceptibility, environmental factors, and abnormal immune responses, in which inflammatory cytokines and chemokines substantially contribute to disease pathogenesis.^[^ ^43^, ^44^ ^]^ However, the role of signaling pathways and related genes in the progression of colitis and CAC is less appreciated. Here, we show that *Smad4* deprivation enhances the expression of TGF‐*β*1 and YAP, and YAP in turn interacts with Smad2/3 and exacerbates the development of colitis and CAC (**Figure** **8**).

Previous studies have proposed a role of TGF‐*β* signaling in gut inflammation and cancer.^[^ ^24^, ^45^ ^]^ TGF‐*β*1, a well‐known cytokine produced by multiple lineages of leukocytes, stromal cells and epithelial cells,^[^ ^46^ ^]^ restrains autoimmune responses by inhibiting differentiation of the Th lymphocytes.^[^ ^47^ ^]^ In line with this, mice bearing T‐cell‐specific T*β*RII abrogation develop systemic autoimmunity that ultimately leads to severe colitis.^[^ ^48^ ^]^ Interestingly, *Smad4* could control T cell's residence in a naïve CD8^+^ state independently of TGF‐*β*, thereby obviating intestinal inflammation.^[^ ^49^ ^]^ Meanwhile, TGF‐*β* signaling via *Smad4* maintains the intestinal barrier function in Lrig1^+^ cells, and its defects are a predisposing factor in IBD and CAC.^[^ ^27^ ^]^ In a recent study, it has been proposed that CCL20/CCR6 signaling involved in mucosal inflammation mediates colitis‐associated colon carcinogenesis induced by *Smad4* loss.^[^ ^28^ ^]^ However, whether the TGF‐*β*/Smad4 axis constrains the development of IBD or CAC in an intestinal epithelium‐intrinsic manner remains unclear. We found that loss of *Smad4* in intestinal epithelial cells increases TGF‐*β*1 expression and lead to exacerbated colitis and CAC in mouse models.

Intestinal epithelial cell lineage specification plays an important role in the intestinal barrier integrity.^[^ ^50^, ^51^, ^52^ ^]^ In agreement with it, we found that *Smad4*‐deficient intestinal organoids display spheroid morphology with compromised secretory cell lineage differentiation upon TGF‐*β*1 exposure. Of particular importance are the reduced mucus‐producing goblet cells and anti‐bacterial Paneth cells that contribute to intestinal inflammation. Therefore, increased TGF‐*β*1 expression may function in two ways in *Smad4*‐deficient colonic epithelium: Breaking down epithelial barrier and inducing inflammation.

The function of YAP/TAZ in the gastrointestinal tract is complicated. While YAP/TAZ is dispensable for the intestinal epithelium in homeostasis,^[^ ^53^ ^]^ it is required for intestinal regeneration upon injury.^[^ ^54^ ^]^ In addition, ubiquitously induced expression of YAP in the mouse intestine leads to epithelial dysplasia,^[^ ^55^ ^]^ whereas it represses metastatic colorectal cancer by reprogramming *Lgr5^+^* cancer stem cells into *Klf6^+^* wound‐healing cells,^[^ ^56^ ^]^ suggesting a dual role of YAP either as an oncogene or a tumor suppressor in a context‐dependent manner. Our study highlights the important role of YAP signaling in mediating TGF‐*β* signaling in the absence of *Smad4*. YAP is upregulated by TGF‐*β*1, and inhibition of YAP activity suppresses the spheroid morphology of TGF‐*β*1‐stimulated *Smad4^−/−^* organoids. It has been proposed that the spheroid shape of intestinal organoids may suggest a reprogramming into a fetal‐like state to maintain the capacity for regeneration upon damage.^[^ ^57^, ^58^, ^59^ ^]^ A recent study also reported that intestinal tumor cells, when exposed to transient TGF‐*β*, can revert to an embryonic state in a YAP/TAZ dependent manner and acquire Wnt‐independent growth.^[^ ^60^ ^]^ We also observed that many fetal genes were upregulated in *Smad4*‐deficient organoids after TGF‐*β* stimulation (data not shown). As a downstream mediator of TGF‐*β* signaling, YAP interacts with Smad2/3 in the absence of *Smad4*. As YAP can occupy the promoters of the key inflammasome genes and initiate their transcription,^[^ ^61^ ^]^ we speculate that YAP does so by coordinating with Smad2/3 in the *Smad4^−/−^* intestinal epithelium and thereby potentiates intestinal inflammation. We found that TAZ interacts with Smad2/3 and this interaction is enhanced following TGF‐*β* stimulation, indicating that TAZ could also act downstream of TGF‐*β* signaling and mediate TGF‐*β* effect in *Smad4*‐deficient intestinal epithelial cells. Indeed, TAZ in human embryonic stem cells controls Smad nucleocytoplasmic translocalization and couples it to the transcriptional machinery.^[^ ^62^ ^]^ It has been suggested that YAP/TAZ sequesters Smad complexes in response to high cell density, thereby suppressing TGF‐*β* signaling.^[^ ^63^ ^]^ TGF‐*β*1 has been reported to activate YAP signaling by remodeling the epithelial cytoskeleton and thus sensing cellular mechanical forces,^[^ ^64^ ^]^ however, how YAP is regulated by TGF‐*β* signaling in *Smad4*‐deficient intestinal epithelial cells remains unknown. Nonetheless, our results indicate that attenuation of YAP/TAZ activity through heterozygous deletion of YAP/TAZ ameliorates intestinal inflammation and impedes the development of colitis. These results open the possibility that partial blockade of YAP activity may alleviate colitis and lower the risk of CAC in IBD patients bearing *Smad4* mutations.

Another open question is about the original cellular source of TGF‐*β*1. We speculate that both epithelial cells and mesenchymal cells can secrete TGF‐*β*1 as evidenced by an increased number of TGF‐*β*1^+^ZO‐1^+^ cells and F4/80^+^TGF‐*β*1^+^ cells after *Smad4* loss. It is likely that activated YAP could drive TGF‐*β*1 expression in intestinal epithelial cells through transcriptional machinery as YAP can activate TGF‐*β* expression in heart pacemaker cells.^[^ ^65^ ^]^ However, we could not rule out that TGF‐*β*1 may come from related immune cells, and both epithelial and immune cells contribute to the enhanced TGF‐*β*1 level in *Smad4* KO mice. Indeed, in several types of cancer, a TGF‐*β*1‐rich tumor microenvironment is maintained by some immune cells, and Tregs, and macrophages, and platelets can serve as the primary TGF‐*β*1 producers.^[^ ^37^ ^]^ Consistently, we observed an elevated expression of TGF‐*β*1 in CD45^+^ leucocytes and F4/80^+^ macrophages after *Smad4* deletion during colitis, although it could be a secondary response to the injured epithelial after DSS treatment. Furthermore, we detected upregulated expression of F4/80 and IFN‐*γ* in inflammatory areas in *Smad4*‐deficient mice, which possibly reflects the early inflammatory environment of colitis in *Smad4*‐deficient mice. High TGF‐*β*1 may affect the function of other immune cells and cause pro‐inflammatory response, depending on the presence of other cytokines, its local concentration, or the type of immune cells it targets.^[^ ^66^ ^]^

In summary, our work demonstrates a potential link between the epithelial *Smad4* status and aberrant immune responses in the intestine and highlights an epithelial‐intrinsic role of TGF‐*β*/Smad4 in colitis and CAC. These findings offer conceptually novel insights into the development and pathogenesis of IBD and CAC by revealing the role of the TGF‐*β*/Smad4 disruption in shaping the immune response, which have therapeutic implications on IBD and colitis‐associated tumorigenesis.

## 4 Experimental Section

### Mice

*Lgr5‐EGFP‐IRES‐CreERT2* ^[^ ^67^ ^]^ mice were obtained from Jackson Laboratory, *Villin^CreER^* ^[^ ^68^ ^]^ mice were a gift from Dr. Sylvie Robine. *Smad4^fl/fl^* ^[^ ^69^ ^]^ mice from Dr. Xiao Yang, and *Yap ^fl/fl^*;*Taz ^fl/fl^* mice from Dr. Dawang Zhou. All mouse strains were bred and housed at the animal facility under specific pathogen free conditions in accordance with institutional guidelines and ethical regulations at the Guangzhou Institutes of Biomedicine and Health, Chinese Academy of Sciences (SYXK2022‐0063). All mice used in this study were generated on a C57BL/6 genetic background and were 2 to 3 months old, unless otherwise indicated. All experiments were conducted using littermates separated at weaning and were in accordance with the national guidelines for housing and care of laboratory animals.

### Generation of Intestinal Epithelial Cell‐Specific Smad4‐Deficient Mice

To generate inducible epithelium‐specific *Smad4* knockout mice, *Smad4^fl/fl^* mice were crossed with *Villin^CreER^* mice, which express Cre recombinase under the control of the villin promoter. Gene knockout was achieved by intraperitoneal injection of tamoxifen (T5648, Sigma‐Aldrich) in corn oil at a concentration of 20 mg mL^−1^ for 5 consecutive days. Mouse genotype was validated at different time points from 7 to 30 days after tamoxifen injection. *Smad4^fl/fl^* mice without Cre recombinase were used as the control in all experiments. The following PCR primers (5’–3’) were used for genotyping WT, *Smad4^fl/+^*, and *Smad4^fl/fl^* mice: Forward primer, GGGCAGCGTAGCATATAAGA; Reverse primer, GACCCAAACGTCACCTTCAC.

### Construction of Colitis and Colitis‐Associated Cancer Model

To induce acute experimental colitis, mice aged 8–10 weeks‐old were given 3.5% DSS (MW 36–50 KDa; MP Biomedicals) dissolved in their drinking water for 5 consecutive days, followed by regular water until sacrificed at the designated time points. The severity of colitis was assessed daily by monitoring relevant parameters, including body weight, posture, and stool. The DAI scores were recorded based on the following criteria: Weight loss (0 for no change, 1 for 5–10%, 2 for 10–15%), and 3 for >15%), body posture (0 for smooth fur without a hunchback, 1 for mild fur and hunchback, 2 for moderate fur and hunchback, and 3 for severe fur and heavy hunchback), and stool consistency (0 for normal, 1 for mild loose stool, 2 for loose stool and diarrhea, and 3 for bloody stools). The colon was isolated from euthanized mice to analyze its length, immune cell ratio and histological score.

For colitic cancer induction, mice were intraperitoneally injected with a single dose of AOM (10 mg kg^−1^; Sigma). 5 days later, 2.5% DSS was added to drinking water for 5 consecutive days, followed by drinking water for 2 weeks. This DSS‐administration cycle was repeated for two additional time courses, and mice were sacrificed 2 weeks after the last DSS cycle. During the experiment, the body weight and survival of mice were monitored. Finally, tumor number, size, and malignancy were assessed after sacrificed.

### Isolation of Intestinal Epithelial Cells and Culture of Organoids

Mouse intestines were isolated and longitudinally cut with cold PBS washing for three times. The villi were entirely removed, and small 1 cm pieces of the intestine were incubated in 2 mm EDTA in PBS at 4 °C for 30 min. The pieces were then rigorously suspended in cold PBS, and the mixture was filtered through a 70 mm cell strainer (BD Biosciences) for purification. The crypt fraction was enriched through centrifugation at a speed of 400–500 × *g* for 3 min, and then embedded in Matrigel (BD Biosciences) and seeded on a 24‐well plate in ENR culture medium (Advanced DMEM/F12 supplemented with Penicillin/Streptomycin, GlutaMAX‐I, N2, B27 and N‐acetylcysteine) containing EGF (50 ng mL^−1^, Invitrogen), Noggin (100 ng mL^−1^, R&D), and R‐spondin1 (500 ng mL^−1^, R&D), as previously described.^[^ ^70^ ^]^

### Fluorescence Activated Cell Sorting Analysis and Isolation of Colonic Immune Cells

Organoids derived from the small intestine of *Lgr5‐EGFP‐IRES‐CreERT2* mice were incubated with TrypLE (Invitrogen) for 15 min to prepare single‐cell suspensions. The dissociated cells were then passed through a 40 µm cell strainer (BD), and single Lgr5‐EGFP^+^ cells were sorted and analyzed by flow cytometry (BD Biosciences, San Diego, CA). To evaluate the ratio of immune cell infiltration in the colon, the remaining colon tissues were collected after the epithelium was removed and digested for 45 min at 37 °C using RPMI‐1640 containing collagenase IV (2.5 mg mL^−1^; Sigma‐Aldrich), DNase I (10 U mL^−1^; Roche), and 3% fetal bovine serum. Single‐cell suspensions were obtained by grinding through a 70 µm cell strainer (BD). The cell suspensions were then centrifuged over Percoll density (GE Healthcare), and immune cells were separated by collecting the interface fractions between 40 and 80% Percoll. After several washes, single‐cell suspensions were stained with anti‐CD45, anti‐CD4, anti‐CD8, anti‐CD11b, anti‐F4/80, anti‐Gr1, and anti‐TGF‐*β*1 at 1:100 dilutions for fluorescence activated cell sorting (FACS) analysis. The single‐cells were incubated with the indicated antibodies for 30 min at 4 °C in the dark. The unlabeled antibodies were washed away, and BD Fortessa (BD Biosciences, San Diego, CA) and FlowJo software were used for data collection and analysis. For colonic macrophage sorting, single‐cell suspensions were stained with anti‐CD45, anti‐CD11b, and anti‐F4/80. Macrophages (CD45^+^CD11b^+^F4/80^+^) were sorted on BD FACSAria Fusion.

### Reverse Transcription and Quantitative Polymerase Chain Reaction Analysis

Total RNA was extracted and purified using TRIZOL reagent (Thermo Fisher Scientific). Equal amounts (1 µg) of total RNA were reverse transcribed to cDNA using HiScript II Q Select RT SuperMix (Vazyme). After reverse transcription, cDNA was amplified by real‐time PCR using ChamQ SYBR Color qPCR Master Mix (Vazyme) according to the manufacturer's instructions. Relative mRNA levels were calculated using the 2‐ΔΔCq method and normalized to GAPDH mRNA. Statistical analysis was performed using Student's *t*‐test, and a *p*‐value <0.05 was considered significant. The quantitative PCR primers were listed in Table S2, Supporting Information.

### Immunoblotting

The intestinal epithelium of mice with colitis and colitic cancer, intestinal organoids, or HEK293T was lysed using a lysis buffer comprising 150 mm NaCl, 10 mm tris (pH 7.4), 5 mm EDTA, 1 mm EGTA, and 1% Triton X‐100, supplemented with protease inhibitors (Roche). The experiments were conducted as described previously.^[^ ^71^ ^]^

### Immunoprecipitation

Proteins were extracted from HEK293T and intestinal organoids using ice‐cold lysis buffer containing 20 mm Tris (PH 7.5), 150 mm NaCl, 1 mm EDTA, 1 mm EGTA, 2.5 mm sodium orthovanadate, 50 mm sodium fluoride, 1% Triton X‐100, and protease inhibitors. The extracts were centrifuged at 15 000 × *g* for 15 min at 4 °C. The resulting supernatants were collected and pre‐cleared with protein A/G beads at 4 °C for 1 h. Next, 5 µg primary antibody or isotype IgG was added to the cleared cell extracts and incubated overnight at 4 °C. Protein A/G beads were then added to the supernatants and incubated at 4 °C for 4 h. The beads were washed three times with washing buffer containing 20 mm Tris‐HCl (pH 7.5), 150 mm NaCl, 1 mm EDTA, 1 mm EGTA, 0.5% TritonX‐100, 2.5 mm sodium pyrophosphate, 1 mm *β*‐glycerophosphate, 1 mm Na3VO4, and protease inhibitors. The bound proteins were eluted by adding 1x SDS loading buffer and heated at 98 °C for 10 min. The eluted proteins were analyzed by immunoblotting.

### Immunofluorescence Staining and Immunohistochemistry

Tissue sections from the intestine were incubated overnight at 4 °C with primary antibodies, followed by incubation with Alexa Fluor‐labeled secondary antibodies for 1 h at room temperature. Nuclei were stained with 4′,6‐diamidino‐2‐phenylindole (DAPI) (Sigma‐Aldrich) and slides were washed with PBS, dried, and mounted using Antifade Mounting Medium (Invitrogen). For immunohistochemistry, formalin‐fixed, paraffin‐embedded sections (5 mm) were deparaffinized in xylene and in decreasing concentrations of alcohols, followed by antigen retrieval, quenching with 0.3% H_2_O_2_, and blocking with 3% BSA in PBS containing 0.1% Triton X‐100 for 60 min. Sections were then incubated overnight at 4 °C with the indicated antibody, followed by addition of a secondary horseradish peroxidase‐conjugated antibody (Invitrogen, 1:200) for 1 h and diaminobenzidine (DAB) chromogen according to the manufacturer's instructions. Finally, slides were counterstained with Mayer's haematoxylin (abs9214, Absin), dehydrated, and mounted with neutral balsam. Slides were visualized and captured using the Zeiss LSM 800 META microscope, and images were processed using ImageJ or Photoshop software. All images shown here were representatives of at least three randomly selected views.

### TUNEL Staining

The mouse intestine was washed with iced PBS and fixed overnight at 4 °C in 4% paraformaldehyde. After dehydration and processing, intestinal tissues were embedded in paraffin. Freshly cryopreserved intestinal “Swiss‐rolls” embedded in paraffin were used to prepare 5 µm thick sections. These sections were deparaffinized and subjected to TUNEL staining according to the manufacturer's instructions using the in situ cell death detection kit (Roche), followed by DAPI co‐staining as described above. Images were acquired using a Zeiss LSM 800 microscope equipped with a 10× objective.

### Chromatin Immunoprecipitation and ChIP‐seq Analysis

Approximately 3 × 10^7^ cells from intestinal organoids or colonic epithelium were harvested and cross‐linked using 3 mm ethylene glycolbis (succinimidylsuccinate) for 30 min at room temperature with shaking, followed by 1% (wt/vol) formaldehyde for 10–15 min at room temperature with shaking. The cross‐linking was stopped with a final concentration of 150 mm glycine for 5 min at room temperature with shaking, and the cells were subsequently washed twice with ice‐cold 1xPBS, lysed at 4 °C for 15 min in lysis Buffer A (50 mm HEPES‐KOH, 140 mm NaCl, 1 mm EDTA, 10% Glycerol, 0.5% NP‐40, 0.25% Triton X‐100) with protease inhibitors (Roche) and centrifuge at 1400 × *g* for 5 min at 4 °C. The nuclear pellet was suspended in 0.2 mL Buffer B (1% SDS, 50 mm Tris‐Cl, 10 mm EDTA, protease inhibitors) for over 30 min on ice. The lysed cells were then sonicated with a Bioruptor (Diagenode) to obtain chromatin fragments (≈150–500 bp) and centrifuged at 13 500 rpm for 5 min at 4 °C. The soluble chromatin was diluted to 1 mL in a tube using ChIP buffer (50 mm HEPES‐KOH, 500 mm NaCl, 1 mm EDTA, 0.1% sodium deoxycholate, 0.5% NP‐40, 0.5% Triton X‐100, protease inhibitors) and added to 5 µg of antibody with rotation at 4 °C overnight. The next day, 40 µL of magnetic protein G beads (Thermo Fisher) was added and incubated for 2 h at 4 °C with rotation. After washing 3 times, the samples were treated with elute buffer (50 mm Tris‐Cl, pH 8.0, 10 mm EDTA, 1% SDS) at 65 °C for 4 h, then RNase A, and Proteinase K, and the cross‐links were reversed 1 h or overnight at 50 °C. Finally, the precipitated DNA was purified using the QIAamp DNA Mini Kit and quantified using the Qubit 2000 (Invitrogen) and Bioanalyzer 1000 (Agilent). Libraries for Illumina sequencing were generated following the TruePrep DNA Library Prep Kit V2 (Vazyme) protocol. A total of 10 cycles were used for PCR amplification for the generation of ChIP‐seq libraries. Amplified ChIP DNA was purified to retain fragments (≈200–500 bp) and quantified using the Qubit 2000 and Bioanalyzer 1000 before multiplexing. For the ChIP‐qPCR assay, 10% of the chromatin extract was reserved for input. To normalize all ChIP signals, the IP efficiency was calculated using the equation: Percent Input = 10% × 2^(C(T) 10%Input Sample‐C(T) IP Sample)^, where the input sample and IP sample were compared.

### RNA‐seq Library Construction and Data Processing

RNA was extracted using Trizol reagent (15 596 026, Thermo Fisher Scientific), and the RNA integrity was assessed by electrophoresis. First‐strand cDNA was synthesized using random hexamer primers and M‐MuLV Reverse Transcriptase (RNase H), followed by synthesis of a second‐strand cDNA using DNA Polymerase I and RNase H. Tailing Mix and RNA Index Adapters were added to the end of the cDNA synthesis reaction. The cDNA fragments were amplified by PCR, and the products were quantified using the Qubit 2.0 Fluorometer (Life Technologies, Grand Island, NY) and the 2100 Bioanalyzer (Agilent). The products were then denatured and circularized, and the single‐strand circular DNA was used to generate the final library. Sequencing was performed on the Illumina platform with a PE150 strategy by Novogene Bioinformatics Technology Co., Ltd (Beijing, China), based on the required effective library concentration and data amount. The depth ranged from 13.7 to 16.8 million reads per sample on a NovaSeq 6000 platform. mRNA expression analysis was performed using Hisat (version 2.1.0) and Ballgown (version 2.20.0). The differentially expressed genes were identified using EdgeR (version 3.30.3) and DESeq2 (version 1.28.1) software. GO and KEGG enrichment analyses were performed using ClusterProfiler (version 3.16.1), while GSEA analysis was performed using the GSEA (version 4.0.3) software.

### Statistical Analysis

Cohort data were collected from GEO (NCBI) and analyzed using R‐Studio software and related R language for normalization, calculation of gene expression, and annotation. The data were presented in column graphs as means ± SD. Unless otherwise specified, statistical significance was calculated using a two‐tailed unpaired Student's *t*‐test for two groups. The statistical analysis was performed with GraphPad Prism 8.0 software, and *p*‐values were showed as *p < 0.05, **p < 0.01, and ***p < 0.001.

## Conflict of Interest

The authors declare no conflict of interest.

## Author Contributions

L.L., Y.W., and S.Y. contributed equally to this work. L.L., Y.W., and Y.‐G.C. conceived the experiments and analyzed the data; L.L. and Y.W. carried out most of the experiments; S.Y. performed the bioinformatics analysis and analyzed the data; Y.L. characterized spheroids morphology; S.H. conducted most RT‐qPCR analysis and ChIP assay. H.L. helped with functional experiments and modified the manuscript; L.L. and Y.‐G.C. wrote the manuscript. All authors contributed to intellectual inputs.

## Supporting information

Supporting Information

## Data Availability

The data that support the findings of this study are available from the corresponding author upon reasonable request.
