# Supplementary material for: Transforming Growth Factor Beta Promotes Inflammation and Tumorigenesis in Smad4‐Deficient Intestinal Epithelium in a YAP‐Dependent Manner
Source: Adv Sci (Weinh). 2023 Jun 1;10(23):2300708. doi: 10.1002/advs.202300708 (PMC10427365; doi:10.1002/advs.202300708)
Supplement: Supplementary file 1 — Supporting Information [file ADVS-10-2300708-s001.pdf]

ADVANCED SCIENCE
Open Access

Supporting Information

for *Adv. Sci.*, DOI 10.1002/advs.202300708

Transforming Growth Factor Beta Promotes Inflammation and Tumorigenesis in Smad4-Deficient Intestinal Epithelium in a YAP-Dependent Manner

*Liansheng Liu, Yalong Wang, Shicheng Yu, Huidong Liu, Yehua Li, Shan Hua and Ye-Guang Chen**

Supplementary information

TGF-β promotes inflammation and tumorigenesis in Smad4-deficient intestinal epithelium in a Yap-dependent manner

Liansheng Liu[1,3†], Yalong Wang[1,3†], Shicheng Yu[1,3†], Huidong Liu[2], Yehua Li[2], Shan Hua[3,4]
Ye-Guang Chen[2,3,4*]

*Corresponding author. Email: ygchen@tsinghua.edu.cn (Y.-G.C.)

This word file includes:

Figures. S1 to S9
Tables S1 to S3

Other Supplementary Materials for this manuscript include the following:

Data files S1 to S2

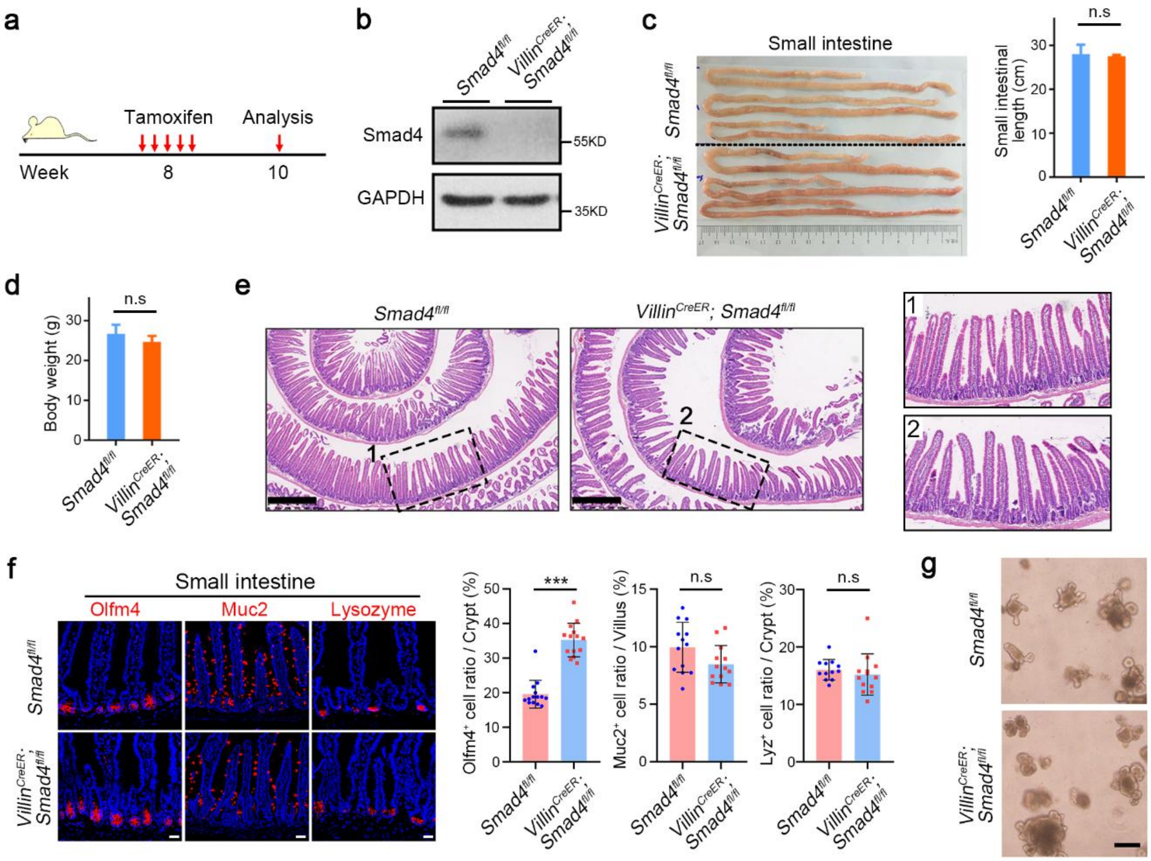

Figure S1. Smad4 deletion in the intestinal epithelium imposes no overt gross phenotypes.

(**a**) Scheme of tamoxifen-induced gene knockout.

(**b**) Anti-Smad4 immunoblotting to verify Smad4 expression in crypts derived from *Smad4$^{fl/fl}$* and *Villin^{CreER};Smad4$^{fl/fl}$* mice.

(**c**) Representative images of the small intestine from epithelial *Smad4*-KO and control littermates, with quantification of the colon length (right). n=3 mice for each group.

(**d**) Quantification of the body weight in epithelial *Smad4*-KO and control littermates. n=3 mice for each group.

(**e**) Representative images of H&E-stained small intestine sections and enlarged field of the indicated sections (right). n=3 mice for each genotype. Scale bars: 625 μm.

(**f**) Immunofluorescence showing the expression of stem cells (Olfm4), goblet cells (Muc2), and Paneth cells (lysozyme) in small intestinal sections, with quantification of the ratios of these cells in the villus-crypt zone. Scale bars: 50 μm.

(**g**) Organoids derived from the small intestine of control littermate *Smad4$^{fl/fl}$* and *Villin^{CreER};Smad4$^{fl/fl}$* mice. Scale bars: 100 μm.

Data are presented as means ± SD with statistical analyses determined by two-tailed Student's *t*-test. ***$p < 0.001$, n.s, no significance.

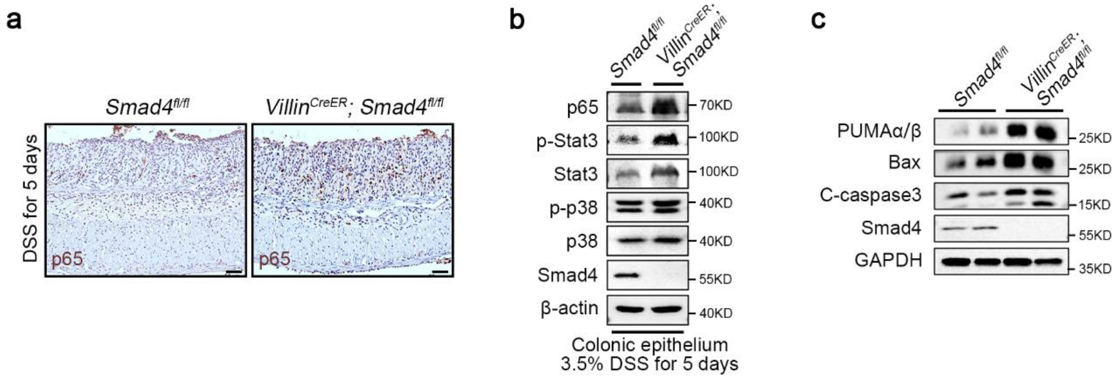

Figure S2. Smad4 loss exacerbates DSS-induced inflammation and disruption of the epithelial barrier.

(**a**) Anti-p65 immunostaining of colon sections from the indicated mice treated with DSS for five days. Scale bar: 50 μm

(**b**) Immunoblotting of colonic epithelium lysates derived from indicated mice after 3.5% DSS treatment for 5 days.

(**c**) Immunoblotting of proliferation- and apoptosis-related proteins in the colonic epithelium from the indicated mice after DSS treatment. n=2 mice for each genotype.

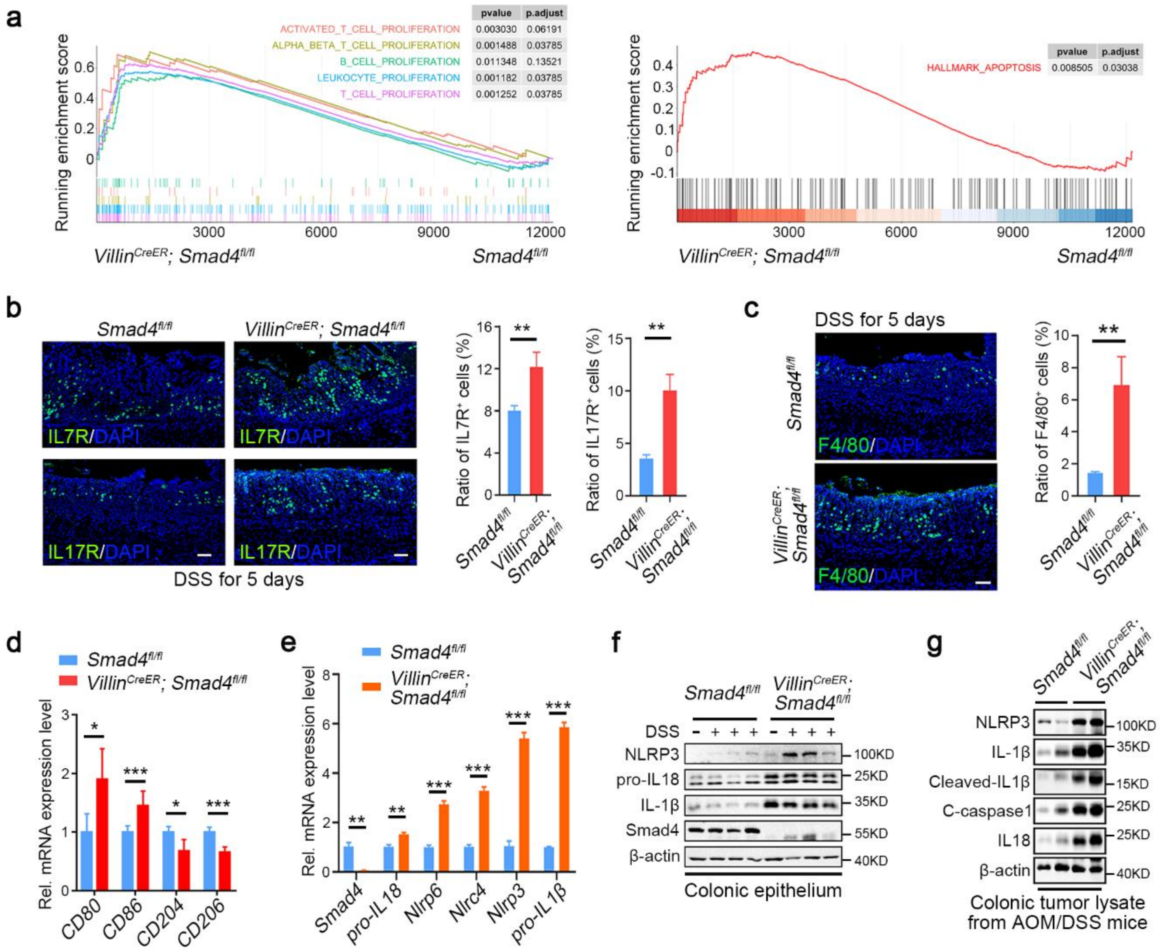

Figure S3. Smad4 deficiency augments immune response.

(**a**) GSEA analysis of the genes related to immune cell proliferation and epithelial cell apoptosis in littermate control *Smad4$^{fl/fl}$* and *Villin^{CreER};Smad4$^{fl/fl}$* mice upon DSS-induced colitis.

(**b**) Immunofluorescence staining of IL-7R and IL-17R in the distal colon derived from DSS-treated mice and statistical analysis of the cell percentage shown in the right (n=3). Scale bar: 50 μm.

(**c**) Immunofluorescence and quantification analysis of F4/80 in the colon sections derived from *Smad4$^{fl/fl}$* and *Villin^{CreER};Smad4$^{fl/fl}$* mice following DSS treatment for 5 days. n=3. Scale bar: 50 μm.

(**d**) RT-qPCR analysis of the markers of M1- and M2-macrophages in colonic tissue derived from *Smad4$^{fl/fl}$* and *Villin^{CreER};Smad4$^{fl/fl}$* mice following DSS treatment for 5 days. n=3 mice for each genotype.

(**e**) RT-qPCR analysis of the indicated inflammasome genes in the colonic epithelium from DSS-treated mice. n=3 mice for each genotype.
(**f**) Immunoblotting of colonic epithelium lysates from the indicated mice with or without DSS treatment. (n=4 mice for each group).
(**g**) Immunoblotting of colonic tumor lysates from the indicated mice challenged with AOM/DSS. (n=2 mice for each group).
Data are presented as means ± SD. Statistical significance is determined by unpaired, two-tailed Student's *t*-test. *$p < 0.05$, **$p < 0.01$, ***$p < 0.001$.

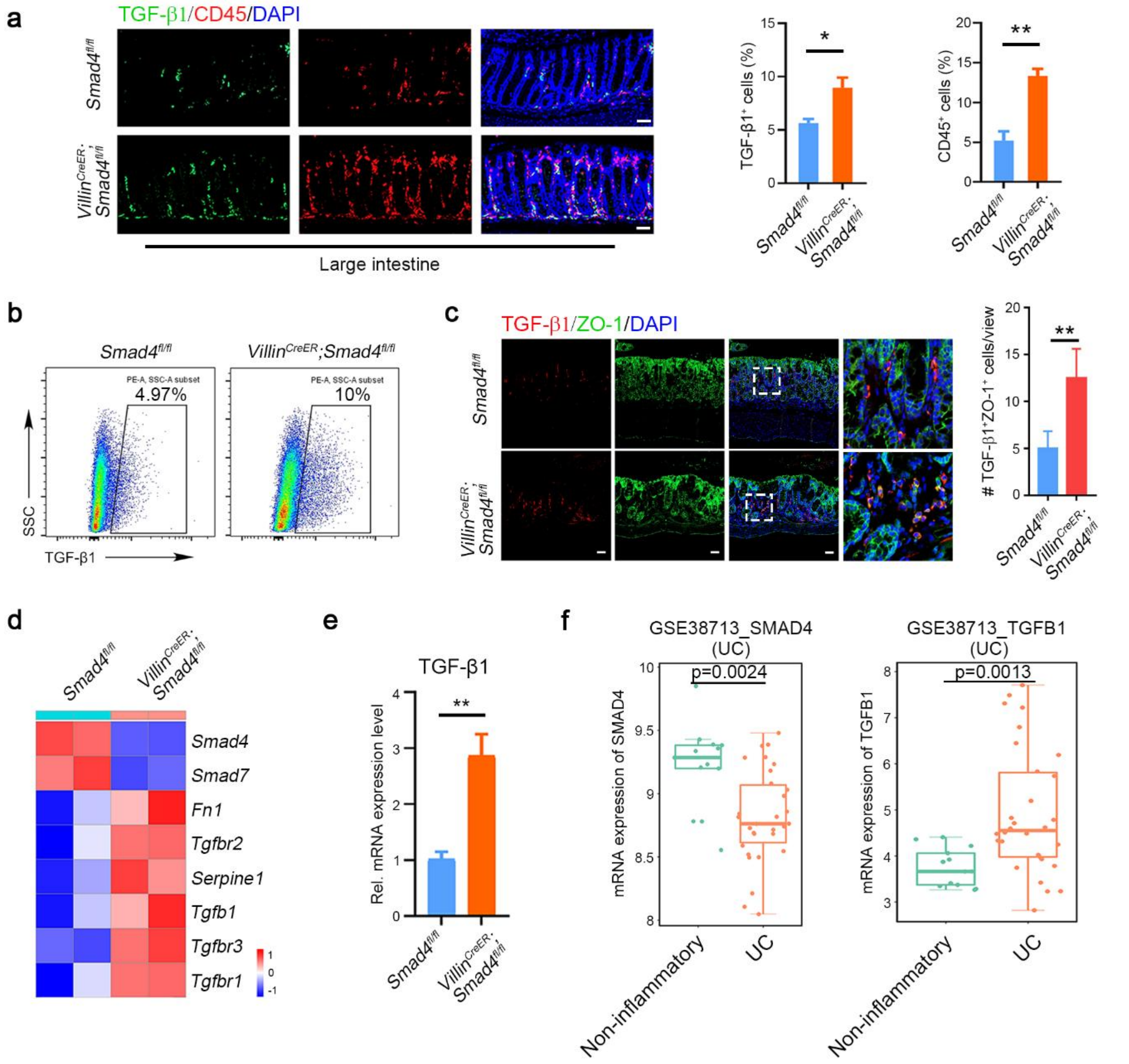

Figure S4. TGF-β is upregulated in *Smad4*$^{-/-}$ colon.

(**a**) Immunofluorescence staining of colon sections with anti-CD45 and anti-TGF-β1 antibodies from the indicated adult mice. Right panel shows the quantification of TGF-β1^{+} and CD45^{+} cells. n=3 mice per group. Scale bar: 50 µm.

(**b**) FACS analysis of colon-infiltrated TGF-β1^{+} cells of DSS-treated littermate control *Smad4*$^{fl/fl}$ and *Villin*^{CreER}*;Smad4*$^{fl/fl}$ mice. SSC, side scatter area.

(**c**) Immunofluorescence co-staining of colon sections with the antibodies against TGF-β1 or tight junction protein ZO-1, and statistics is shown on the right.

(**d**) Heatmap of TGF-β target genes in the indicated mice after DSS treatment. n=2 mice per group.

(**e**) RT-qPCR analysis of TGF-β1 expression in the indicated mice after DSS treatment. n=3 biological replicates.

(**f**) Box plots for *SMAD4* and *TGFB1* expression from the datasets of intestinal biopsies from UC and non-inflammatory individuals (GSE38713).

Data are presented as means ± SD. Statistical significance is determined by unpaired, two-tailed Student's *t*-test. $*p < 0.05$, $**p < 0.01$,

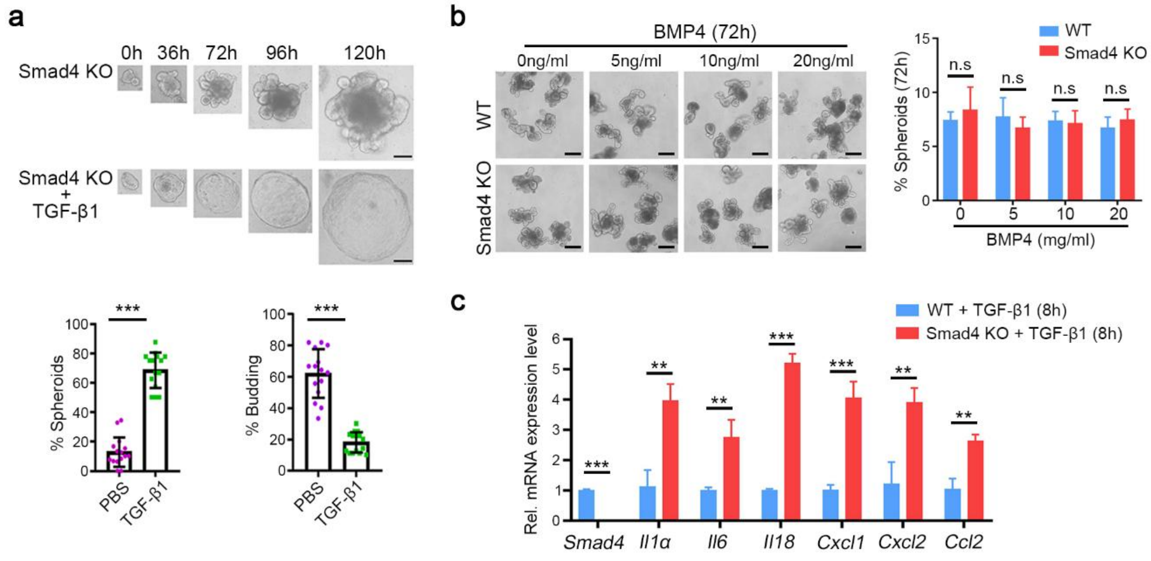

Figure S5. TGF-β induces spheroids and evokes an epithelial-intrinsic immune response in *Smad4$^{-/-}$* organoids.

(**a**) Morphology of *Smad4*-deficient organoids with or without 0.5 nM TGF-β1 treatment. Quantification of spheroids and budding organoids is evaluated (lower panel). Scale bar: 100 μm.

(**b**) Morphology of WT and Smad4-deficient organoids after stimulating with different concentrations of BMP4. Quantification of the ratio of spheroids is shown on the right. Scale bar: 100 μm.

(**c**) RT-qPCR analysis showing gene expression of proinflammatory cytokines and chemokines in the indicated intestinal organoids with TGF-β1 treatment for 8 hours.

Data are presented as means ± SD. Statistical significance is determined by unpaired, two-tailed Student's *t*-test. **$p < 0.01$, ***$p < 0.001$, n.s, no significance.

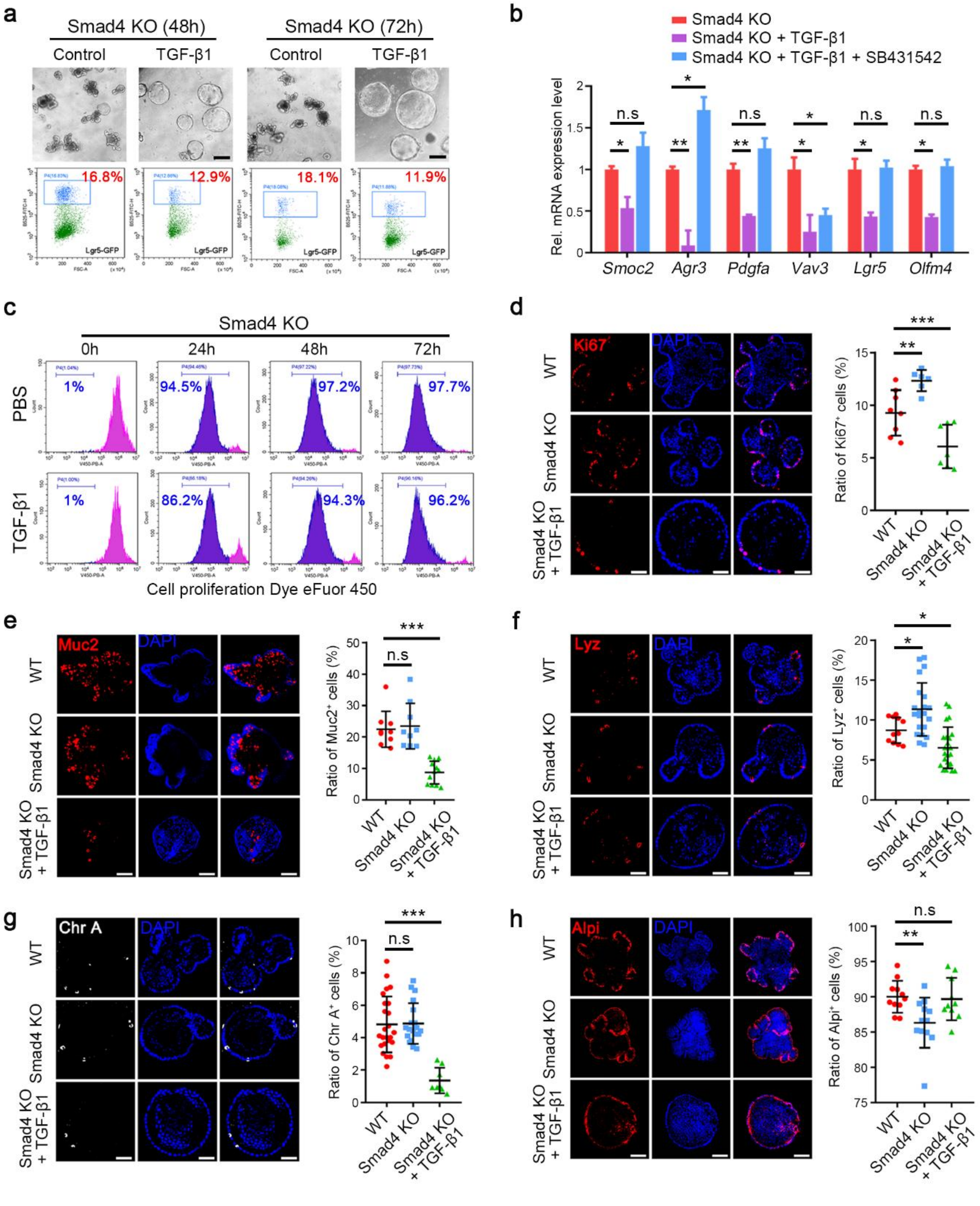

Figure S6. TGF-β1 impairs ISC proliferation and differentiation in Smad4-deficient organoids.

(**a**) Morphological change and FACS analysis of Lgr5-GFP$^+$ cells in *Smad4*-deficient organoids with or without 0.5 nM TGF-β1 stimulation. Scale bar: 100 μm.

(**b**) mRNA expression level of intestinal stem cell markers in the organoids with or without TGF-β1 and TGFβRI inhibitor (SB431542, 10 μM) treatment. n=3 biological replicates.
(**c**) Flow cytometric analysis of cell division upon TGF-β1 stimulation in *Smad4*-deficient organoids using eFlour 450.
(**d**) Ki67 immunostaining of the indicated organoids and quantification of the Ki67 positive cell. Scale bar: 100 μm.
(**e**) Mucin 2 (Muc2) immunostaining and quantification of $Muc2^{+}$ cells. Scale bar: 100 μm.
(**f**) Lysozyme (Lyz) immunostaining and quantification of Lyz^{+} cells. Scale bar: 100 μm.
(**g**) Immunostaining of Chromogranin A (Chr A) counterstained with DAPI. Right panel shows the quantification of $ChrA^{+}$ cells. Scale bar: 100 μm.
(**h**) Alkaline phosphatase (Alpi; Absorptive cells) staining of the indicated organoids with quantification shown in the right. Scale bar: 100 μm.
Data are presented as mean ± S.D. Statistical significance is determined by unpaired, two-tailed Student's *t*-test. *$p < 0.05$, **$p < 0.01$, ***$p < 0.001$, n.s, no significance.

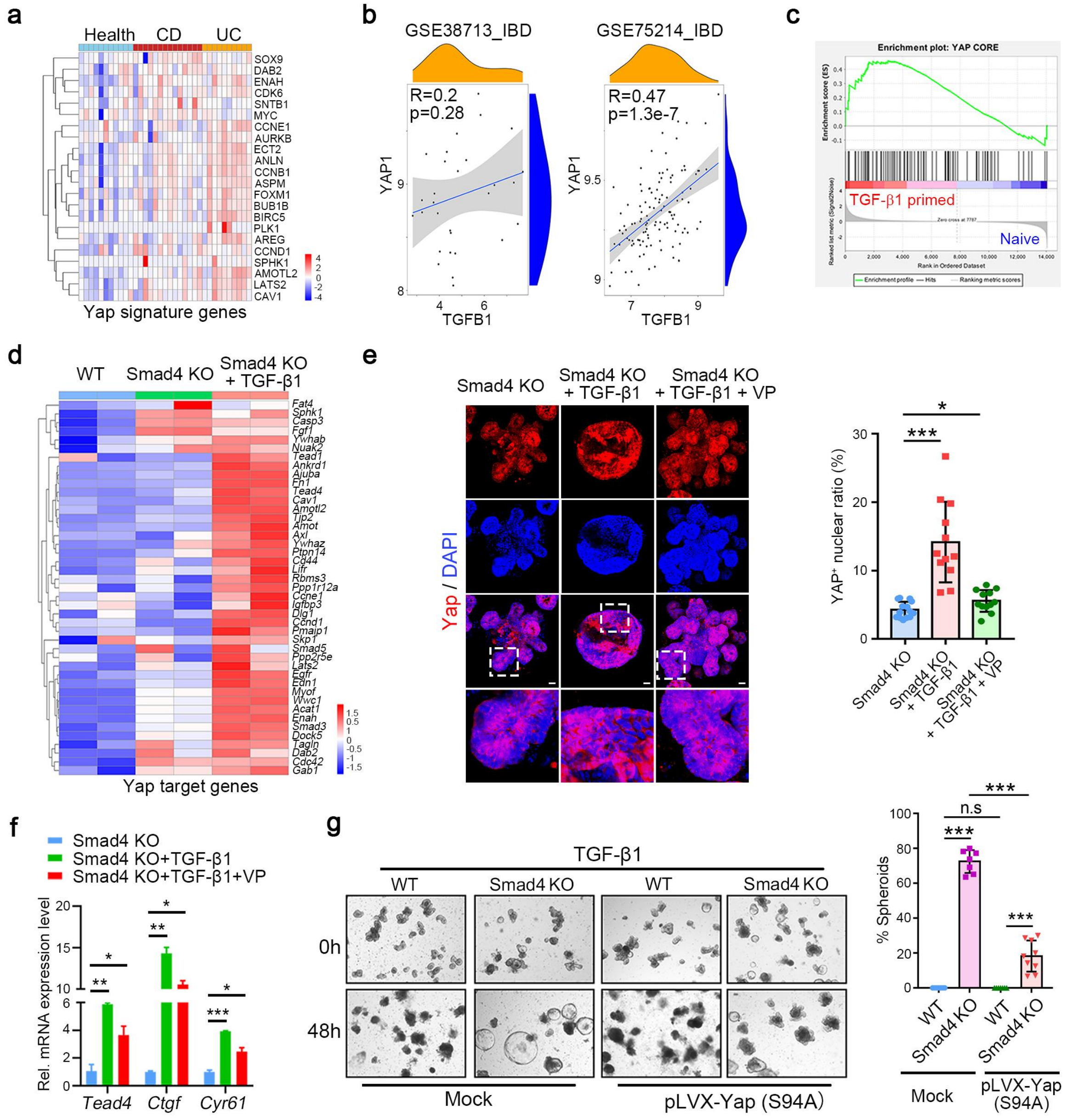

Figure S7. YAP mediates TGF-β effects in Smad4-deficient organoids.

(**a**) Heatmap of the YAP signature genes in IBD patients based on the dataset GSE10616.

(**b**) *In silico* analysis of the indicated online database for *YAP1* and its correlation with *TGFB1* expression in human IBD patients.

(**c**) GSEA of the YAP signature genes from the RNA-seq data derived from *Smad4*-deficient organoids with or without 0.5 nM TGF-β1 treatment.

(**d**) Heatmap of the YAP target genes in the indicated organoids with or without TGF-β1 stimulation. n=2 biological replicates for each group.

(**e**) Immunostaining of YAP (red) indicating its subcellular distribution in indicated organoids with quantification shown on the right. Scale bars: 50 μm.
(**f**) RT-qPCR analysis of the expression of YAP target genes in indicated organoids with or without TGF-β1 and VP treatment.
(**g**) Bright field images of WT and *Smad4*-deficient organoids with/without YAP(S94A) overexpression after TGF-β1 treatment. Quantification is shown on the right. n=3 biological replicates for each timepoint. Scale bar: 50 μm.
Data are presented as means ± S.D. Statistical significance is determined by unpaired, two-tailed Student's *t*-test. *$p < 0.05$, **$p < 0.01$, ***$p < 0.001$, n.s, no significance.

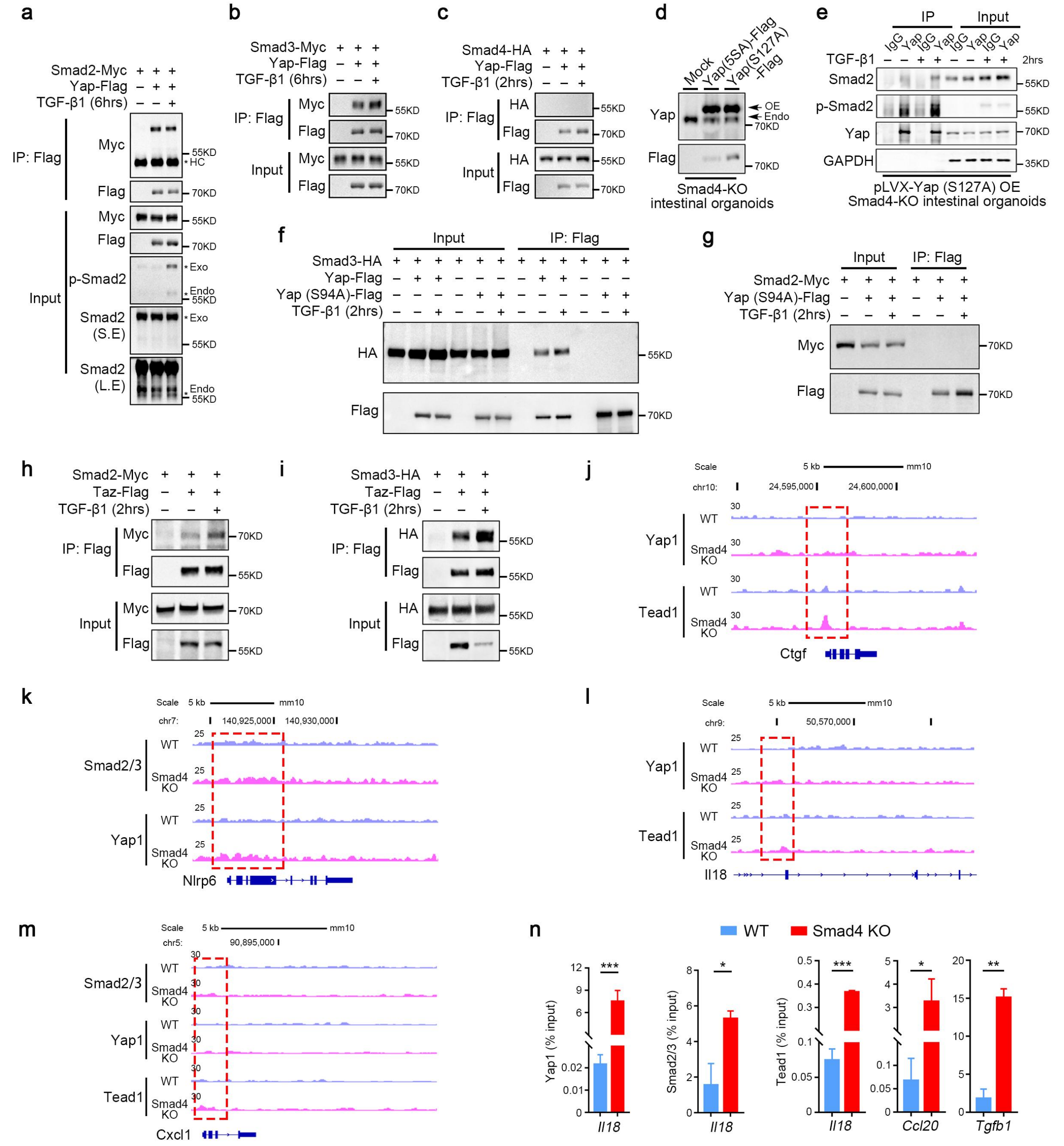

Figure S8. TGF-β enhances the interaction between YAP/TAZ and Smad2/3.

(**a, b, c**) Co-immunoprecipitation (IP) showing the interaction between overexpressed Yap and Smad2/3/4 in HEK-293T cells with or without 0.5 nM TGF-β1 stimulation.

(**d**) Immunoblotting showing YAP(5SA) and YAP(S127A) expression in *Smad4*$^{-/-}$ intestinal organoids.

(**e**) Co-IP analysis of the interaction between overexpressed YAP(S127A) and Smad2 with or without 0.5 nM TGF-β1 treatment in Smad4 KO organoids.

(**f, g**) Co-IP showing that Smad2/3 did not interact with YAP(S94A).
(**h, i**) Exogenous TAZ interacted with Smad2/3, and its interaction was promoteed by 0.5 nM TGF-β1 treatment.
(**j, k, l, m**) Genomic views of Yap1, Smad2/3 and Tead1 ChIP enrichment in the indicated genes in TGF-β1-stimulated organoids.
(**n**) ChIP-qPCR showing Yap1, Smad2/3 and Tead1 occupancy in the indicated genes from colonic epithelium.
Data are presented as means ± S.D. Statistical significance is determined by unpaired, two-tailed Student's *t*-test. *$p < 0.05$, **$p < 0.01$, ***$p < 0.001$.

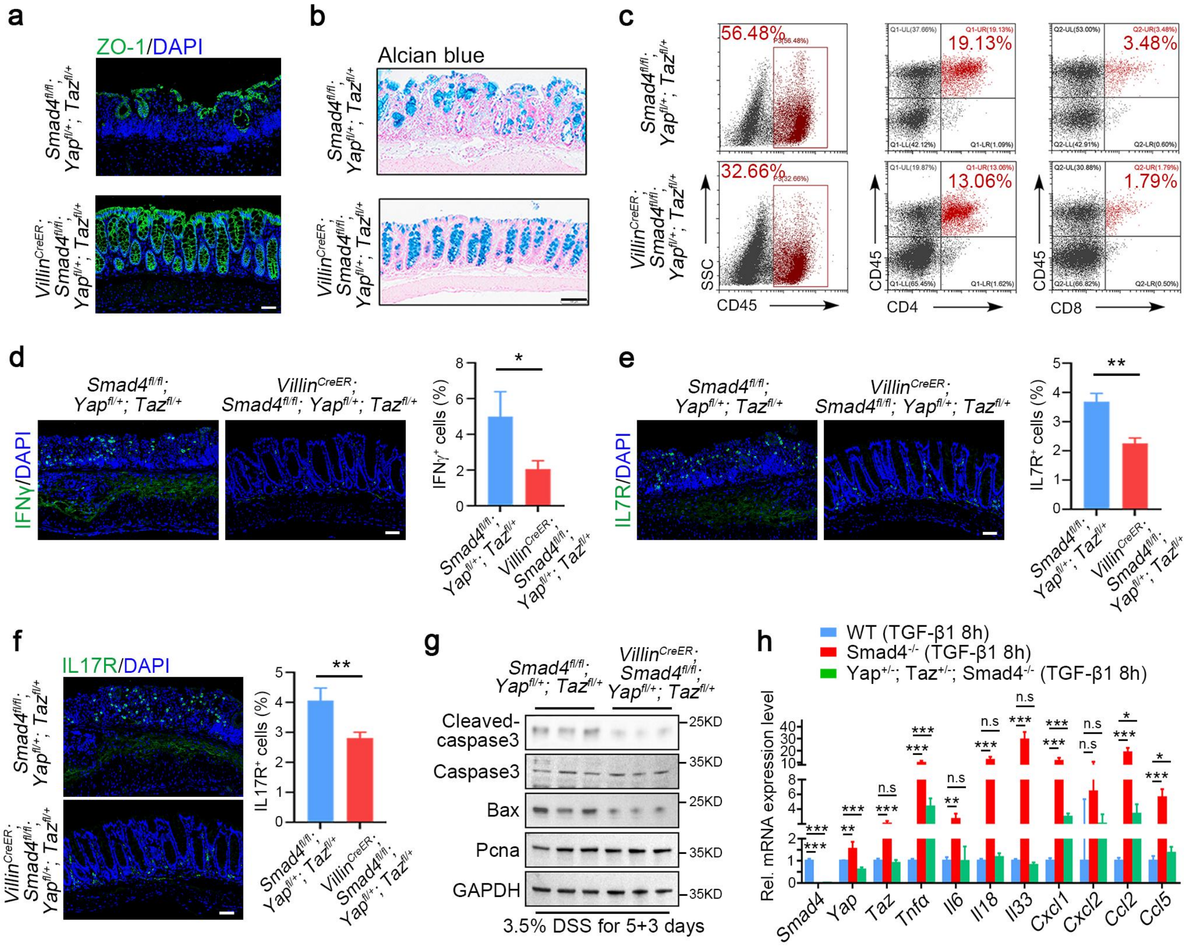

Figure S9. Reducing YAP/TAZ expression attenuates inflammation in *Smad4$^{-/-}$* intestine.

(**a**) ZO-1 immunostaining in the colon of the indicated mice after DSS treatment. n=3 mice each genotype. Scale bars: 50 μm.

(**b**) Alcian Blue-Periodic acid Schiff (AB-PAS) staining of the colon sections from DSS-treated mice. n=3 mice each genotype. Scale bars: 100 μm.

(**c**) Flow cytometric analysis of the colon-infiltrated immune cells from the indicated mice on day 5 after DSS treatment. Data are presented as representative plots.

(**d, e, f**) Immunostaining and quantification of interferon γ^{+} (IFNγ^{+}) cells (**d**), IL7R^{+} cells (**e**), and IL17R^{+} cells (**f**) in the colon of the indicated mice following 5 days of DSS treatment. n=3 mice each genotype. Scale bars: 50 μm.

(**g**) Immunoblotting of apoptosis- and proliferation-related genes in colonic epithelium derived from control and *Smad4$^{-/-}$/Yap$^{+/-}$/Taz$^{+/-}$* mice after DSS treatment. n=3 mice each genotype.

(**h**) RT-qPCR analysis of the expression of proinflammatory cytokines and chemokines in the intestinal organoids with TGF-β1 treatment for 8 hours.

Data are presented as means ± S.D. Statistical significance is determined by unpaired, two-tailed Student's *t*-test. *$p < 0.05$, **$p < 0.01$, ***$p < 0.001$, n.s, no significance.

Table S1. Key resources

REAGENT	SOURCE	IDENTIFIER
Antibodies		
IL17R	Santa Cruz	sc-376374
Smad4	Santa Cruz	sc-7966
F4/80	Santa Cruz	sc-377009
ZO-1	Santa Cruz	sc-33725
IFNγ	Santa Cruz	sc-8423
IL7R	Santa Cruz	sc-514445
Pcna	Santa Cruz	sc-56
Bax	Santa Cruz	sc-7480
PUMAα/β	Santa Cruz	sc-374223
Smad2/3	Abcam	Ab202445
Smad2/3	CST	8685
p-Smad2	CST	18338
IL-1β	CST	31202
Cleaved IL-1β	CST	63124
NLRP3	CST	15101
Caspase-1	CST	24232
Cleaved caspase-1	CST	89332
AIM2	CST	63660
ASC/TMS1	CST	67824
YAP	CST	14074
YAP1	Novus	NB110-58358SS
YAP/TAZ	CST	93622
Olfm4	CST	39141
Caspase3	CST	9662
Cleaved Caspase3	CST	9664
p65	CST	8242
Stat3	CST	9139
p-Stat3	CST	9145
p38	CST	8690
p-p38	CST	4511
Anti-FIAG	CST	14793
Anti-HA	CST	3724
Anti-Myc	CST	2276
TEAD1	ABclonal	A5218
IL-18	Proteintech	10663-1-AP
β-tubulin	Proteintech	10094-1-AP
β-actin	Abcam	ab8226
CD45	Abcam	ab40763
MUC2	Abcam	ab272692
Lysozyme	Abcam	ab108508
GAPDH	Abcam	ab8245

Ki67	Abcam	ab15580
TGF-β1	This paper	Chen lab
Chemicals and Reagents		
Dextran Sulfate Sodium, DSS	MP Biomedicals	9011-18-1
Azoxymethane, AOM	Sigma-Aldrich	A5486
Trizol	Thermo Fisher	15596026
HiScript II Q Select RT SuperMix	Vazyme	R232-01
ChamQ SYBR Color qPCR Master Mix	Vazyme	Q431-02
TrypLE	Invitrogen	12604021
4′,6-diamidino-2-phenylindole (DAPI)	Sigma-Aldrich	28718-90-3
Antifade Mounting Medium	Invitrogen	P36930
in situ cell death detection kit	Roche	12156792910
Protease inhibitor Cocktail	Biotool	B14001
DAB	Sangon Biotech	DB0140
EDTA Antigen Retrieval	Sangon Biotech	E673003
Opti-MEM	Gibco	31985-070
Advanced DMEM/F-12	Gibco	12634028
N-2	Gibco	A1370701
B-27	Gibco	17504044
Matrigel	R&D	BME001-05
GlutaMAX	Gibco	35050061

Table S2. List of Primer Pairs Used for Quantitative Polymerase Chain Reaction Analysis in this Study

Gene	Forward	Reverse
Il6	GCCAGAGTCCTTCAGAGAGA	GGTCTTGGTCCTTAGCCACT
Tnfa	CCCTCACACTCAGATCATCTTCT	GCTACGACGTGGGCTACAG
Il-1b	TACCTGTGTCTTTCCCGTGG	TTGTTCATCTCGGAGCCTGT
Tgfβ1	CTCCCGTGGCTTCTAGTGC	GCCTTAGTTTGGACAGGATCTG
Il18	GTCTACCCTCTCCTGTAAGAACA	TGGCAAGCAAGAAAGTGTCC
Nlrp6	CACACCCAGAATGAGACCAG	GTAGCCATAAGCAGCTCCCT
Nlrc4	CTACATTGATGCTGCCTTGG	ATCCGTCACTGCTCACACAG
Nlrp3	ATTACCCGCCCGAGAAAGG	TCGCAGCAAAGATCCACACAG
Smad4	CAGCCATAGTGAAGGACTGTTGC	CCTACTTCCAGTCCAGGTGGTA
Tead4	GCTCTGGATGTTGGAGTTCTCG	TTGGGCTTGACTGGCTGATGTG
Ctgf	GGGCCTCTTCTGCGATTTC	ATCCAGGCAAGTGCATTGGTA
Cyr61	CTGCGCTAAACAACTCAACGA	GCAGATCCCTTTCAGAGCGG
Agr2	GCAGTTTGTTCTCCTCAACCTGG	GTATCGTCCAGTGATGTCTGCC
Muc2	CTACCACCATTACCACCACTAC	GTCTCTCGATCACCACCATTT
Tff3	TCCAAGCCAATGTATGGTGCCG	CAGGGCACATTTGGGATACTGG
Mmp7	AGGTGTGGAGTGCCAGATGTTG	CCACTACGATCCGAGGTAAGTC

Lyz1	ACGAGCTACAAACTACAACCG	GATCTCTCACCACCCTCTTTG
Reg4	CTGGCTATCAGAGAAACCTGCC	CTGGCTTCACTCTTTGTCCTGG
Lct	ACGCTGGATGACACGGAAAGGA	CCACTCGAAGTTGTCCATCAGC
Alpi	GTCCCACCGCTGGTTACTTT	CTGTGGGCTGAGATGATGTC
Cbr1	CCTTCCACATTCAAGCAGAGGTG	CTGAGACTCACCATGCTGGACA
Smoc2	GCCAAGTGCAAAGATCCACAGC	ACACTTGCTGGAACTCCTTCCG
Agr3	TTCACCACCTGGAGGACTGTCA	GACAAGTTCTTGTCCGTGGTCTC
Pdgfa	CTGGCTCGAAGTCAGATCCACA	GACTTGTCTCCAAGGCATCCTC
Vav3	GACCAATGGACTTCGGAGAGCT	CTGCCTGGATATGCAATGGTGG
Lgr5	CGTAGGCAACCCTTCTCTTATC	GCACCATTCAAAGTCAGTGTTC
Olfm4	CGAGACTATCGGATTCGCTATG	TTGTAGGCAGCCAGAGGGAG
pro-IL-1b	CTCCATGAGCTTTGTACAAGG	TGCTGATGTACCAGTTGGGG
pro-Il18	ACTGTACAACCGCAGTAATACGG	AGTGAACATTACAGATTTATCCC
GAPDH	AGGTCGGTGTGAACGGATTTG	TGTAGACCATGTAGTTGAGGTCA
CD80	CCTCAAGTTTCCATGTCCAAGGC	GAGGAGAGTTGTAACGGCAAGG
CD86	ACGTATTGGAAGGAGATTACAGCT	TCTGTCAGCGTTACTATCCCGC
CD204	CGCACGTTCAATGACAGCATCC	GCAAACACAAGGAGGTAGAGAGC
CD206	GTTCACCTGGAGTGATGGTTCTC	AGGACATGCCAGGGTCACCTTT

Il1α	ACGGCTGAGTTTCAGTGAGACC	CACTCTGGTAGGTGTAAGGTGC
Cxcl1	TCCAGAGCTTGAAGGTGTTGCC	AACCAAGGGAGCTTCAGGGTCA
Cxcl2	CATCCAGAGCTTGAGTGTGACG	GGCTTCAGGGTCAAGGCAAACT
Ccl2	GCTACAAGAGGATCACCAGCAG	GTCTGGACCCATTCCTTCTTGG
Yap	CCAGACGACTTCCTCAACAGTG	GCATCTCCTTCCAGTGTGCCAA
Taz	GTCACCAACAGTAGCTCAGATCC	GTTGCTGAGGAAGTCTTCTGGAG
Il33	CTACTGCATGAGACTCCGTTCTG	AGAATCCCGTGGATAGGCAGAG
Ccl5	CCTGCTGCTTTGCCTACCTCTC	ACACACTTGGCGGTTCCTTCGA

Table S3. List of Primer Pairs Used for Chromatin Immunoprecipitation-Quantitative Polymerase Chain Reaction Analysis in this Study

Gene	Forward	Reverse
Yap1	GCTTGAAGAAGGAGTCGGGC	CTTCAATGCCGTCATGAACCC
Il18	GTATCACAGGCACAGCTGGA	CTGATGCTGGAGGTTGCAGA
Ccl20	TTGTGGTGACAGGATGAGGC	GGGAATGTACACAAGAAGGCG
Tgfb1	CCTCTTGAGTCCCTCGCATC	GTGAGGCTCTGACACCAAGG

Data files S1. Gene expression of colonic epithelial cells upon colitis by RNA-Seq analysis.

Data files S2. Gene expression by RNA-seq in small intestinal organoids.
